# Multi-Omics Mendelian Randomization Maps Lipid-Related Brain Cell-Type Associations with Kidney Disease

**DOI:** 10.3390/genes17091097

**Published:** 2026-09-11

**Authors:** Jiani Deng, Boning Cao, Fengjie Zheng, Sinan Ai, Yaoxian Wang, Xu Wang

**Affiliations:** 1 Dongzhimen Hospital of Beijing University of Chinese Medicine, Beijing 100700, China; jenny19940527@gmail.com(J.D.); zhengfengjie@bucm.edu.cn (F.Z.); 2 School of Traditional Chinese Medicine, Beijing University of Chinese Medicine, Beijing 100029, China; 3 Diabetes Department of Integrated Chinese and Western Medicine, China-Japan Friendship Hospital, Beijing 100029, China; aisinan223@163.com; 4 Nephrology Diagnosis and Treatment Center, The First Affiliated Hospital of Henan University of Chinese Medicine, Zhengzhou 450000, China; 5 Department of Nephropathy, The First Affiliated Hospital of Henan University of Chinese Medicine, Zhengzhou 450000, China; 6 Department of Nephrology, China-Japan Friendship Hospital, Beijing 100029, China

**Keywords:** Mendelian randomization, brain–kidney axis, single-cell eQTL, lipid metabolism, Bayesian colocalization

## Abstract

**Background:** Cellular heterogeneity limits causal inference in complex diseases. We applied an established cell-type-stratified Mendelian randomization (csMR) framework, originally developed by Hao et al. (2024), to investigate whether lipid phenotypes affect kidney disease through distinct brain cell populations, extended by a proteome-wide mediation component. **Methods:** Using Bayesian colocalization (posterior probability of hypothesis 4 [PPH4], ≥0.8) and multidimensional instrumental variable selection, we evaluated cell-type-stratified MR-supported associations of five lipid traits across ten brain cell or tissue strata with kidney disease risk. Instrumental variables were derived from published genome-wide association studies and human brain single-cell expression quantitative trait locus (eQTL) maps. An exploratory proteomic mediation analysis using the Difference Method was performed with UKB-PPP as the discovery resource and deCODE as the external validation resource, with mediation signals interpreted as hypothesis-generating. For the primary csMR analysis, multiple testing was corrected separately within each lipid trait using a Bonferroni threshold of 3.33 × 10^−4^, corresponding to 150 cell/tissue-by-outcome tests per lipid trait. **Results:** In csMR analyses, cholesterol-related lipids showed cell-type-stratified associations with kidney disease, most prominently in oligodendrocyte-related analyses (max β on the log-odds scale = 1.025). LDL-related associations with broad chronic glomerular disease were most evident in excitatory neuron-related analyses. Dual-cohort proteomic analysis prioritized plasma proteins, including SNAP29 and ICAM4, as candidate protein-associated signals for exploratory mediation analysis. These findings represent genetic colocalization and Mendelian randomization-supported associations, not experimentally established mechanisms, and should be interpreted as hypothesis-generating. **Conclusions:** This study provides a cell-type-resolved genetic and proteomic association map linking lipid traits, brain cell strata, and kidney disease outcomes, offering hypotheses for future experimental validation.

## 1. Introduction

The central nervous system (CNS) acts as a pivotal terminal for systemic homeostasis, coordinating multidimensional physiological networks through neuroendocrine feedback and autonomic regulation [1,2,3,4]. While the hypothalamic-pituitary-adrenal (HPA) axis is well-established in metabolic regulation [5,6], emerging evidence suggests a more complex “brain-peripheral” crosstalk that extends beyond classical endocrine pathways. Genetic studies have increasingly linked cerebral lipid homeostasis to systemic pathologies; however, the relationship between brain-related genetic regulatory signals and extra-cerebral metabolic diseases, particularly kidney disease, remains poorly understood.

A major challenge in deciphering these cross-organ communications is cellular heterogeneity. Traditional bulk-tissue analyses mask the distinct genetic architectures of diverse brain cell types, such as neurons, astrocytes, and microglia [7,8]. Recent advances in single-cell RNA sequencing (scRNA-seq) and cell-type-specific expression quantitative trait locus (eQTL) mapping have revealed that nearly half of expression-regulating loci (eGenes) exhibit cell-type-dependent effects. This suggests that disease-associated variants identified in genome-wide association studies (GWAS) likely exert their pathogenic effects through specific cellular subpopulations rather than the tissue at large.

Multiple lines of evidence indicate that distinct brain cell types are involved in systemic metabolic and renal pathophysiology. First, central neuroinflammation and sympathetic overactivity are established risk factors for kidney disease, with astrocytes and microglia increasingly recognized as key regulators of these processes [9]. Second, oligodendrocytes have a uniquely high cholesterol demand for myelination, coupling central lipid metabolism to whole-body energy balance via pathways such as the GPR17–cAMP–PKA axis; dysregulation of this axis has been linked to both metabolic syndrome and neuroinflammation [10,11]. Third, excitatory and inhibitory neuronal populations—particularly those in the paraventricular nucleus (PVN) and the dorsal motor nucleus of the vagus—directly control renal sympathetic and vagal output, thereby affecting glomerular hemodynamics and electrolyte handling [12]. Fourth, endothelial cells and pericytes of the neurovascular unit serve as gatekeepers of blood–brain barrier transport and have been implicated in the cerebral microvascular dysfunction that accompanies chronic kidney disease [13]. This background evidence motivated the inclusion of endothelial cells and pericytes in the cell-type-stratified analysis, but the present genetic analyses did not directly measure blood–brain barrier transport or neurovascular signaling. Despite this accumulating biological evidence, systematic population-level causal inference linking specific brain cell types to kidney disease via genetically anchored approaches has not been performed.

Mendelian randomization (MR) has emerged as a robust framework for causal inference by utilizing genetic variants as instrumental variables, thereby minimizing confounding and reverse causation [14]. Nevertheless, conventional MR relies on tissue-level data, which cannot resolve the cell-layer mechanisms through which genetic risks are manifested. While previous MR studies have examined the relationship between circulating lipids and kidney outcomes, they treated the brain as a black box, ignoring the cell-type-specific regulatory landscape through which lipid-associated genetic signals may be annotated to brain cell populations. A systematic, cell-resolved causal framework for the lipid-mediated brain–kidney axis is therefore still lacking.

How brain cell-type-related genetic regulation may be linked to peripheral renal outcomes remains unclear. Circulating proteins provide measurable systemic molecular traits that may help prioritize candidate cross-organ pathways. However, in the present study, proteomic analyses were used for exploratory statistical mediation rather than to prove direct brain-to-plasma signaling or intercellular communication [15,16]. Recent proteogenomic studies have demonstrated that genetically predicted protein levels can identify novel drug targets and causal biomarkers for complex diseases [17,18]. However, most current models lack the resolution to link specific brain cell types to the systemic proteome. Integrating cell-specific eQTLs with large-scale proteomic data (e.g., UK Biobank and deCODE) offers an opportunity to map the brain-to-plasma signaling axis, providing a granular view of how cerebral lipid metabolism may influence the circulating environment and, ultimately, renal integrity.

In this study, we applied an established csMR framework to examine whether lipid-associated genetic signals annotated to human brain cell types are associated with renal outcomes. By integrating brain single-cell eQTL maps, GWAS summary statistics, and circulating proteomic data, we generated a cell-type-stratified genetic and proteomic association framework linking lipid traits, brain cell strata, plasma proteins, and kidney disease outcomes. These findings prioritize candidate brain cell strata and protein signatures for future validation, but do not establish direct brain-to-kidney communication.

## 2. Materials and Methods

### 2.1. Overview of csMR Framework

**Figure 1:**
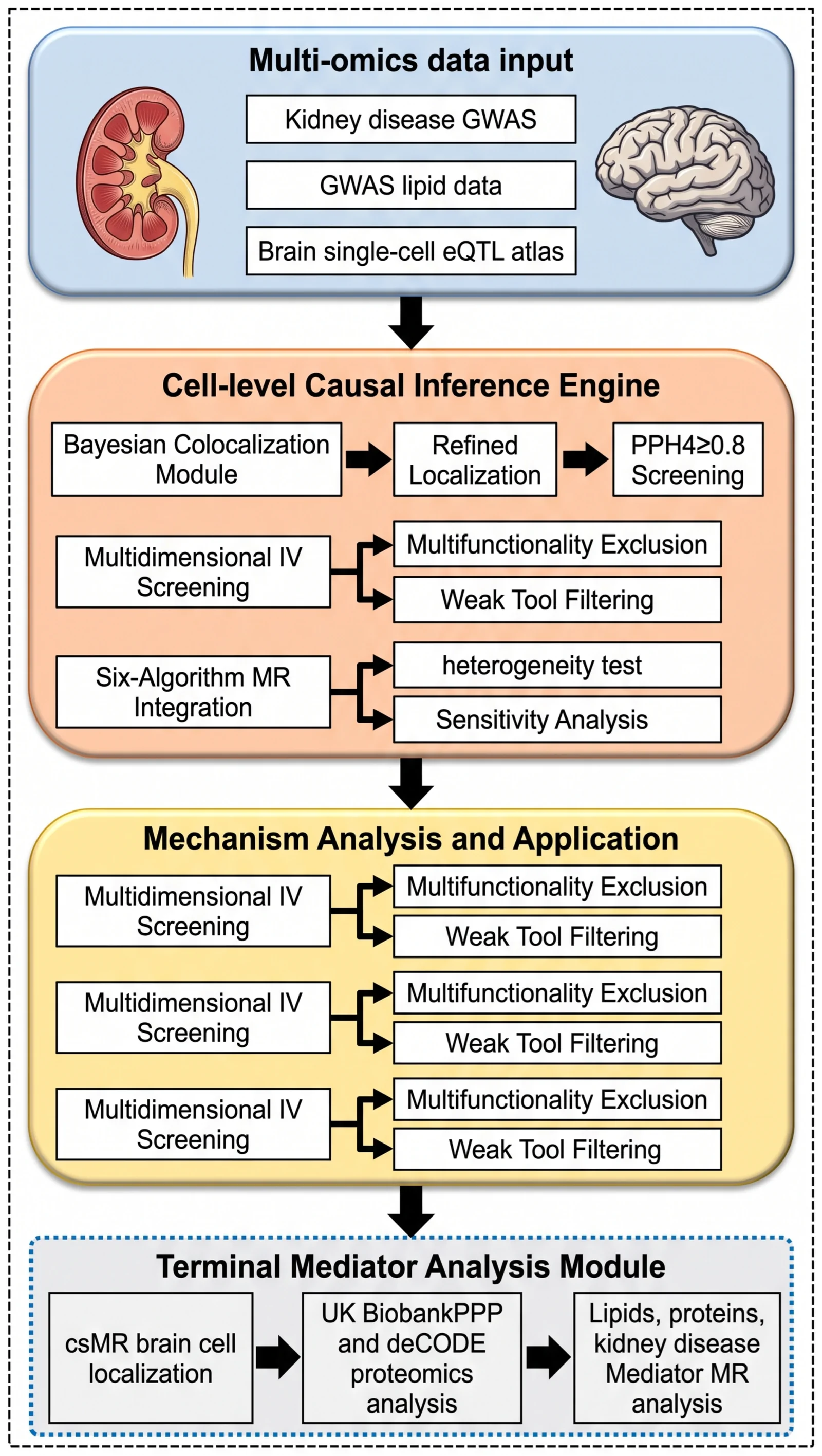
Process Framework Diagram. PPH4: posterior probability of hypothesis 4 in Bayesian colocalization; IV: instrumental variable; IVW: inverse variance weighting; MR: Mendelian randomization; eQTL: expression quantitative trait locus; GWAS: genome-wide association study. All abbreviations are defined at first use in the text.

To dissect cell-level genetic regulatory mechanisms, we applied a cell-type-stratified Mendelian randomization (csMR) framework originally developed by Hao et al. (2024) [19]. The methodology implements a hierarchical three-tier inference system (Figure 1): (1) variant fine-mapping and genetic colocalization, employing the SuSiE algorithm (R package "susieR", version 0.14.2, https://github.com/stephenslab/susieR, accessed on 18 January 2026) [20] and the Coloc Bayesian framework (R package "coloc", version 5.2.3, https://github.com/chr1swallace/coloc, accessed on 18 January 2026) to identify shared causal variants between GWAS and single-cell eQTL (sc-eQTL) data [7,21,22]; (2) instrumental variable (IV) refinement, involving multidimensional filtering to select high-confidence genetic instruments; and (3) multi-algorithm causal estimation, to quantify the causal effects of exposures on kidney-related outcomes across discrete cell populations. The csMR computational platform is open-source and available on GitHub (https://github.com/rhhao/csMR, accessed on 18 January 2026). A detailed process diagram is provided in Figure 1.

### 2.2. Dataset Sources

#### 2.2.1. Single-Cell and Bulk eQTL Data

We integrated brain sc-eQTL summary statistics from Bryois et al. (2020) [7], encompassing eight major brain cell types: excitatory neurons (ExN), inhibitory neurons (InN), astrocytes, microglia, oligodendrocytes (ODC), oligodendrocyte precursor cells (OPC), endothelial cells (EC), and pericytes. The dataset comprises 14,595 genes and approximately 5.3 million single-nucleotide polymorphisms (SNPs) derived from the prefrontal and temporal cortices of 192 individuals of European ancestry. For comparative analysis, bulk-tissue brain eQTL data were obtained from the Genotype-Tissue Expression (GTEx) project version 8 [23], restricted to cortex and frontal cortex (BA9). Source eQTL data were first checked for genome-build annotation and were subsequently harmonized with other datasets to the final analytical genome build, GRCh37/hg19. Table 1 summarizes the characteristics of the integrated eQTL datasets.

We applied the previously established csMR framework of Hao et al. to a new biological question: whether lipid traits influence kidney disease through specific human brain cell populations. Compared with conventional two-sample MR, which usually treats genetic instruments at the trait level, and bulk eQTL-informed MR approaches, which rely on tissue-level regulatory effects, csMR uses cell-type-specific eQTL information to assign genetic regulatory effects to discrete brain cell populations. The main methodological extension in the present study was the integration of this cell-stratified causal framework with two large-scale plasma proteomic resources, UK Biobank Pharma Proteomics Project and deCODE, to explore candidate lipid–brain cell–plasma protein–kidney disease mediation pathways.

In reporting colocalization results, unique lead SNPs refer to near-independent lipid-associated variants before colocalization, whereas cell-type-stratified colocalization counts refer to SNP–cell-type or gene–cell-type events summed across brain cell strata unless otherwise specified.

#### 2.2.2. GWAS Summary Statistics

Several GWAS and proteomic resources used in this study may include UK Biobank participants. Therefore, we performed a dataset-level assessment of potential sample overlap among lipid GWAS, renal-outcome GWAS, and plasma-proteomic datasets. For each dataset, cohort source, ancestry, sample size, GWAS identifier, and possible UK Biobank contribution were checked according to the original publication or database documentation. Datasets were classified as having no known overlap, possible overlap, or likely overlap with UK Biobank-derived resources. The full assessment is provided in Table S7.

Because individual-level data were not available, overlapping participants could not be directly excluded. To reduce potential bias from sample overlap and winner’s curse, we prioritized strong genetic instruments with F-statistics > 10, applied the same prespecified instrument-selection rules across analyses, and interpreted associations based on small instrument sets cautiously. Single-SNP Wald-ratio estimates were labeled as provisional. For proteomic analyses, the UK Biobank Pharma Proteomics Project (UKB-PPP) was used as the discovery cohort and deCODE was used as an external validation cohort. Findings supported only by potentially overlapping datasets were interpreted as hypothesis-generating.

We systematically searched publicly available GWAS summary statistics for lipid traits and kidney-related outcomes. The search was performed through three major repositories: (i) the IEU Open GWAS platform (https://gwas.mrcieu.ac.uk/ accessed on 18 January 2026), (ii) the GWAS Catalog (https://www.ebi.ac.uk/gwas/, accessed on 18 January 2026), and (iii) FinnGen Release 10. Dataset selection followed pre-specified criteria: (a) population of European ancestry (≥80%), (b) sample size ≥ 5000, (c) non-overlapping discovery cohorts across datasets to avoid sample duplication, and (d) availability of full summary statistics.

Lipid traits. Five lipid phenotypes—total cholesterol (TC), low-density lipoprotein cholesterol (LDL-C), high-density lipoprotein cholesterol (HDL-C), triglycerides (TG), and very low-density lipoprotein cholesterol (VLDL-C)—were selected as exposures. Summary statistics were obtained from the UK Biobank (Neale Lab; N ≈ 340,000–360,000) and the Global Lipids Genetics Consortium (GLGC) [24].

Kidney outcomes. Fifteen kidney disease outcomes were included, covering the major categories: (1) glomerular diseases (chronic glomerulonephritis, IgA nephropathy, membranous nephropathy, FSGS, membranoproliferative GN, minimal change disease, lupus nephritis, diabetic nephropathy), (2) hypertensive nephropathy, (3) CKD (broad definition), (4) ESKD, (5) AKI, (6) kidney neoplasms (kidney cancer, renal cell carcinoma), and (7) kidney cyst. Data sources included FinnGen R10, UK Biobank, and publicly available GWAS meta-analyses.

Because chronic glomerulonephritis (CGN) appeared repeatedly in the downstream analyses, we provided additional phenotype-level information for this outcome. CGN was analyzed using the GWAS identifier GCST90018820. The phenotype was treated as a broad chronic glomerular disease outcome rather than a single histopathological subtype. Available information on phenotype name, GWAS identifier, source database, ancestry, sample size, case/control counts, diagnostic-code basis, genome build, and possible participant overlap is summarized in Table S7. Where case/control counts or diagnostic codes were not available from the summary-level documentation, these fields were explicitly marked as not reported rather than inferred. Therefore, all CGN-related downstream interpretations were restricted to broad chronic glomerular disease and were not interpreted as subtype-specific mechanisms.

To ensure consistency across eQTL, GWAS, and LD reference datasets, all variants used for downstream colocalization, and Mendelian randomization analyses were harmonized to GRCh37/hg19 coordinates. When source datasets were originally reported in GRCh38/hg38, genomic coordinates were converted to GRCh37/hg19 using a standard liftOver procedure. Variants that could not be uniquely mapped, mapped to multiple genomic locations, lacked rsID annotation, or showed inconsistent chromosome-position information after conversion were excluded.

Linkage disequilibrium estimation and clumping were performed using the European ancestry reference panel from the 1000 Genomes Project, matched to GRCh37/hg19 coordinates. After coordinate harmonization, variants were further checked for allele concordance across exposure, eQTL, proteomic, and outcome datasets. SNPs with mismatched effect alleles, unresolved strand ambiguity, incompatible allele frequencies, duplicate identifiers, or missing effect estimates were removed before downstream analysis. Palindromic variants with ambiguous allele frequencies were excluded unless strand orientation could be reliably inferred. The final harmonized SNP sets were then used for colocalization, instrumental-variable selection, and MR analyses.

Assessment of sample overlap and winner’s-curse bias. Several GWAS and proteomic resources used in this study may include UK Biobank participants. We therefore performed a dataset-level assessment of possible participant overlap among lipid GWAS, renal-outcome GWAS, and plasma-proteomic datasets. Each dataset was classified according to the source description as having likely UK Biobank overlap, no known UK Biobank overlap, or unclear overlap. The complete assessment is provided in Table S7.

Individual-level data were unavailable; therefore, overlapping participants could not be directly removed. To reduce potential bias from sample overlap and winner’s curse, we prioritized strong instruments with F-statistics > 10, applied consistent instrument-selection rules across analyses, distinguished single-instrument Wald-ratio findings from multi-instrument MR results, and interpreted small-instrument or potentially overlapping analyses cautiously. For proteomic analyses, UK Biobank Pharma Proteomics Project (UKB-PPP) was used as the discovery dataset, whereas deCODE was used as an external validation dataset (Table 2).

**Table 2:** Detailed characteristics of GWAS summary statistics for lipid-related traits.

Trait	GWAS ID	Sample Size	Population	Source/Consortium
**Total Cholesterol**	ieu-b-109	437,878	European	UK Biobank/GLGC
**VLDL Cholesterol**	met-d-VLDL_C	115,082	European	Kettunen et al.
**HDL Cholesterol**	ieu-b-108	403,943	European	UK Biobank/GLGC
**LDL Cholesterol**	ieu-b-110	440,546	European	UK Biobank/GLGC
**Triglycerides**	ieu-b-111	441,016	European	UK Biobank/GLGC

### 2.3. Causal Inference Analysis and Co-Localization

We employed the Sum of Single Effects (SuSiE) variational Bayesian inference model for fine mapping of genetic variants, combined with the Bayesian colocalization (Coloc) framework to evaluate the posterior probability of each hypothesis. The primary metric for assessing shared causal variants between eQTLs and GWAS signals was the posterior probability of hypothesis 4 (PPH4), defined as the probability that a single shared causal variant is associated with both traits. A stringent threshold of PPH4 > 0.8 was applied to identify significant colocalization signals. The highly polymorphic MHC region (chr6: 28,477,797–33,448,354, GRCh37) was excluded to prevent spurious associations.

This integrated approach offers three advantages: (1) improved locus prioritization by incorporating SuSiE-derived fine-mapping information for loci with multiple causal signals; (2) stringent pleiotropy control through systematic filtering; and (3) cell-resolved functional annotation enabling the mapping of systemic traits to discrete cellular regulatory pathways.

Full colocalization results with posterior probability of colocalization (PPH4 ≥ 0.8), including GWAS trait, eQTL region, colocalized gene, brain cell type, lead SNPs, and posterior probabilities, are provided in Table S1.

### 2.4. Selection and Refinement of Genetic Instrumental Variables (IVs)

IVs selection followed the three core MR assumptions:

Relevance: Candidate IVs were identified at genome-wide significance (*p* < 5 × 10^−8^). Instrument strength was evaluated using F-statistics, and instruments with F > 10 were retained to reduce weak-instrument bias, which is particularly relevant when potential sample overlap exists between exposure and outcome datasets.

Independence: Independent genetic signals were identified via Linkage Disequilibrium (LD) clumping (PLINK v1.9; *r*^2^ < 0.001, window = 10,000 kb) [25]. Confounding phenotypes including BMI, smoking status, or hypertension -were filtered using PhenoScanner (v2.0) database with an exclusion threshold of *p* < 5 ×10^−5^ [26,27].

Exclusion Restriction and Data Harmonization: During data harmonization via the Two Sample MR (v0.5.6) R package [28], palindromic SNPs with intermediate allele frequencies (MAF > 0.42) and strand inconsistencies were removed to eliminate orientation ambiguity. For SNPs missing in the outcome dataset, proxies with *r*^2^ > 0.8 were utilized. After PhenoScanner filtering, the remaining SNPs were used as genetic instruments for downstream MR analysis. PhenoScanner screening was used to reduce the influence of variants associated with measured potential confounders, but it should not be interpreted as proof that the MR independence or exclusion-restriction assumptions were fully satisfied. These assumptions were further evaluated indirectly through pleiotropy and heterogeneity sensitivity analyses.

### 2.5. Quality Control and Instrumental Variable (IV) Refinement

To ensure the reliability of the genetic instrumental variables (IVs), we implemented a multidimensional quality control pipeline via the Radial MR (v1.0) framework [29]. Horizontal and directional pleiotropy were systematically evaluated using a two-stage testing model.

Pleiotropy Detection: Cochran’s Q test and Rücker’s Q test were used to identify outliers. Outlier and heterogeneity assessment: Heterogeneity and potential outlying instruments were assessed using Cochran’s Q test, Rücker’s Q test, Radial MR, and MR-PRESSO. These analyses were prespecified and applied consistently across all lipid traits and renal outcomes. Outlier detection was used as a sensitivity assessment rather than as an iterative procedure to force global heterogeneity tests to become non-significant. The primary MR estimates were based on the prespecified instrument set after genome-wide significance filtering, LD clumping, harmonization, allele-concordance checking, and PhenoScanner screening. When radial or MR-PRESSO outliers were identified, outlier-corrected estimates were reported as secondary sensitivity results and compared with the primary estimates.

Instrument Strength Validation: The collective strength of the refined IV set was quantified using the joint *F*-statistic, calculated as follows:

$$F=\frac{R^{2}(N-K-1)}{k(1-R^{2})}$$

where *R*^2^ = $\frac{\beta^{2}}{\beta^{2}+{SE}^{2}*N}$ represents the variance explained by the IVs. Only IV sets with a global *F* ≥ 10 were retained to mitigate weak instrument bias.

Full sensitivity analyses are provided in Table S4a–e, including MR-PRESSO global tests, MR-Egger intercept tests, Cochran’s Q tests, Rücker’s Q tests, F-statistics, leave-one-out analyses, and available SNP filtering or outlier-assessment information.

### 2.6. Two-Sample Mendelian Randomization (MR) Framework

Sample overlap can bias two-sample MR estimates toward the observational association, especially in the presence of weak instruments, results from potentially overlapping datasets were interpreted together with instrument strength, estimator type, and sensitivity analyses.

We established a multi-algorithmic inference framework using the TwoSampleMR (v0.5.6) package. For associations supported by two or more independent SNPs, inverse-variance weighted (IVW) MR was used as the primary estimator, while MR-Egger, weighted median, MR-RAPS, and weighted mode were used as complementary sensitivity estimators. For associations supported by only one SNP, the Wald-ratio method was used.

Inverse Variance Weighting (IVW): Applied as the primary method using a random-effects model. Complementary estimators and sensitivity analyses: MR-Egger, weighted median, MR-RAPS, and weighted mode were used as complementary estimators for multi-instrument analyses where applicable. MR-PRESSO, MR-Egger intercept tests, Cochran’s Q tests, Rücker’s Q tests, and leave-one-out analyses were used to assess pleiotropy, heterogeneity, and influential instruments [30,31,32]. Robust MR estimators were used to assess the sensitivity of the causal estimates to potential invalid instruments without relying solely on iterative SNP deletion. Specifically, weighted median, MR-RAPS, weighted mode, MR-Egger, and MR-PRESSO were used as complementary analyses under different assumptions regarding horizontal pleiotropy and instrument validity.

Effect-scale definition and numerical reporting: For binary renal outcomes, MR and csMR effect estimates were reported as β values on the log-odds scale. Therefore, the null value for β is 0. Odds ratios were calculated as OR = exp(β) and were reported only when explicitly labeled as OR, with a null value of 1. Effect estimates were interpreted per genetically predicted unit increase in the corresponding lipid trait or protein level, according to the scale of the original GWAS summary statistics. Cell-type-stratified csMR tables report β estimates rather than ORs unless otherwise specified. *p* values displayed as 0 or smaller than machine precision in the raw output were considered computational underflow and were reformatted using lower-bound notation, such as *p* < 1 × 10^−300^.

Multiple-testing framework: For the primary cell-type-stratified MR analysis, the hypothesis family was defined separately for each lipid trait as 15 renal outcomes × 10 cell or tissue strata, resulting in 150 primary cell/tissue-by-outcome tests per lipid exposure. Therefore, the Bonferroni-corrected significance threshold was set at *p* < 0.05/150 = 3.33 × 10^−4^. Correction was applied separately within each lipid trait because total cholesterol, VLDL-C, HDL-C, LDL-C, and triglycerides were considered related but biologically distinct exposure families. IVW was used as the primary MR estimator, whereas MR-Egger, weighted median, MR-RAPS, weighted mode, and Wald ratio were considered sensitivity or alternative estimators rather than additional primary hypothesis families. Colocalization analyses used the predefined PPH4 ≥ 0.8 threshold for variant prioritization, and proteomic and mediation analyses were interpreted as separate downstream analyses with FDR adjustment where applicable.

Conventional two-sample MR results and cell-type-stratified MR results for total cholesterol, VLDL-C, HDL-C, LDL-C, and triglycerides are provided in Table S3a–j. Table S3a,c,e,g,i report conventional MR results, including β estimates, ORs, 95% confidence intervals, *p* values, FDR-adjusted *p* values, and instrument numbers. Table S3b,d,f,h,j report cell-type-stratified csMR results, including β estimates on the log-odds scale, 95% confidence intervals, *p* values, estimator type, number of instruments, lipid traits, kidney outcomes, and brain cell types. Full-precision numerical estimates are provided in the Supplementary Tables to avoid misleading interpretation caused by rounding.

### 2.7. Proteomics-Based Mediation Analysis

The analytical workflow for the proteomics-based mediation analysis consisted of a proteome-wide protein–disease association discovery and validation procedure. Protein–disease associations were first screened in the UK Biobank Pharma Proteomics Project (UKB-PPP) dataset. Proteins that met the discovery-level statistical criterion in UKB-PPP and showed nominal significance with directionally consistent effects in deCODE were considered externally supported candidate protein associations. The subsequent figures summarize this discovery–validation association workflow. These figures should not be interpreted as systematic pQTL-MR analyses or systematic protein–outcome colocalization analyses. Protein–outcome colocalization was not available for all candidate proteins, and multivariable MR was not performed for all protein signals. The subsequent mediation analysis was performed separately using the Difference Method and was interpreted as exploratory.

Full sensitivity analyses for total cholesterol, VLDL-C, HDL-C, LDL-C, and triglycerides are provided in Table S4a–e. These analyses include MR-PRESSO global tests, MR-Egger intercept tests, Cochran’s Q tests, Rücker’s Q tests, F-statistics, and leave-one-out assessments. The cis/trans status of protein instruments was recorded where available and considered when interpreting pleiotropy risk.

To explore whether circulating plasma proteins may statistically mediate lipid–brain cell–kidney disease associations, we performed an exploratory proteomics-based mediation analysis. Plasma protein–disease association summary statistics were obtained from UKB-PPP as the discovery resource and from deCODE as the external validation resource. This step was used to identify externally supported protein associations for for the subsequent mediation analyses, rather than to perform systematic pQTL-MR or protein–outcome colocalization. Candidate proteins were first screened in UKB-PPP, with statistical significance defined as false discovery rate (FDR) < 0.05. Proteins showing nominal significance in deCODE (*p* < 0.05) with directionally consistent effects were considered externally supported candidate proteins [17]. This replication rule was used to balance discovery-level multiplicity control with independent validation, but the resulting mediation findings were interpreted as exploratory because of the large number of proteins and candidate pathways tested.

The Difference Method was used to estimate statistical mediation. For each candidate lipid–brain cell–protein–kidney pathway, the total effect (β_total) was defined as the lipid-to-kidney effect estimated from the primary MR model [33]. The direct effect (β_direct) was defined as the lipid-to-kidney effect after accounting for the candidate protein mediator. The indirect effect was calculated as:

β_indirect = β_total − β_direct

For binary renal outcomes, β estimates were interpreted on the log-odds scale. The standard error of the indirect effect was approximated using the delta method. Assuming approximate independence between the total and direct effect estimates, the variance of the indirect effect was calculated as: Var(β_indirect) = Var(β_total) + Var(β_direct); The 95% confidence interval was calculated as: β_indirect ± 1.96 × SE_indirect; The mediation proportion was calculated as: Mediation proportion = β_indirect/β_total × 100% Mediation proportions were interpreted only when β_total was not close to zero and the total and indirect effects were directionally consistent. Negative mediation proportions or indirect effects in the opposite direction were interpreted as opposite-direction or suppressor-like statistical mediation signals rather than as evidence of definitive biological mediation.

Potential sample overlap among lipid GWAS, renal-outcome GWAS, UKB-PPP, and deCODE was assessed at the dataset level and is summarized in Table S7. Individual-level data were unavailable, so overlapping participants could not be directly removed. Horizontal pleiotropy was assessed using PhenoScanner filtering, MR-Egger intercept tests, MR-PRESSO, heterogeneity tests, and consistency across robust MR estimators where applicable. Because protein–outcome colocalization was not systematically available for all candidate proteins, the mediation results were interpreted as prioritized hypotheses requiring further validation. Lipid–protein association results used for pathway construction are provided in Table S6.

### 2.8. Protein–Protein Interaction and Functional Annotation Analysis

To improve the biological interpretability of the replicated plasma protein signals, we performed exploratory protein–protein interaction and functional annotation analyses. Replicated proteins identified in both the UKB-PPP discovery cohort and the deCODE validation cohort were submitted to STRING-db using Homo sapiens as the reference organism [34]. Functional annotation was performed using Gene Ontology biological process and pathway annotations to summarize major biological themes [35]. These analyses were used only to support biological interpretation and were not considered evidence of experimentally confirmed protein-mediated mechanisms [36].

## 3. Results

### 3.1. Co-Localization of Total Cholesterol with Brain Cell-Type eQTLs

**Figure 2:**
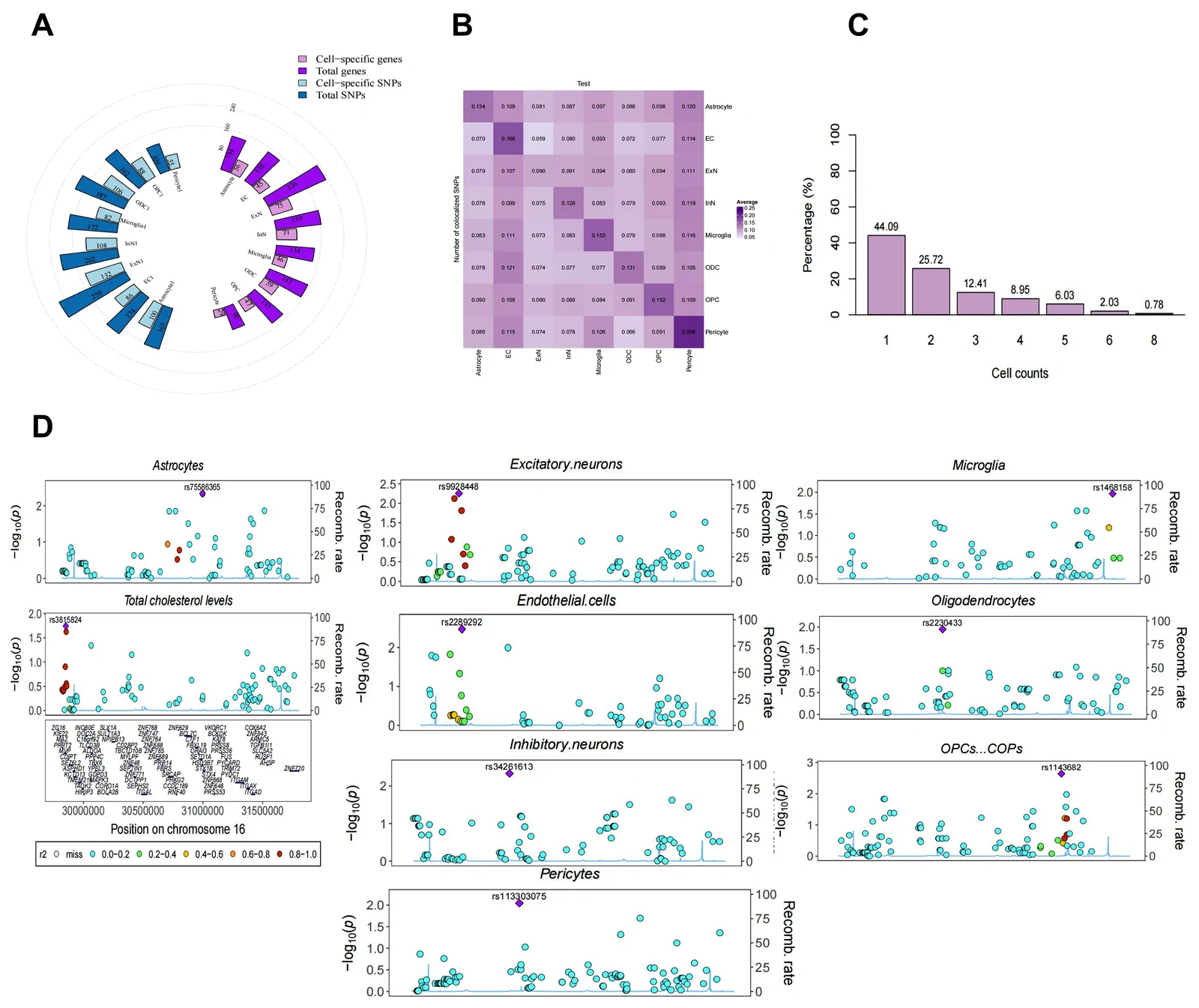
Cell–type–specific colocalization of lipid-associated genetic variants in the human brain. (**A**) Numbers of total and cell–type-specific colocalized SNPs and genes across major brain cell types. (**B**) Cross-cell-type comparison of normalized eQTL effect sizes, showing that colocalized variants often display stronger regulatory effects in their matched cell type. (**C**) Distribution of colocalized SNPs according to the number of brain cell types in which they were detected. (**D**) LocusZoom plots illustrating representative cell-type-specific colocalization signals across astrocytes, endothelial cells, excitatory neurons, inhibitory neurons, microglia, oligodendrocyte lineage cells, oligodendrocytes, and pericytes. This figure summarizes colocalization evidence between lipid-associated GWAS loci and brain cell-type eQTL signals; MR effect estimates are not shown.

Genomic investigation of total cholesterol initially identified 254 near-independent lead single-nucleotide polymorphisms (SNPs) associated with the lipid trait (genome-wide significance threshold *p* < 5 × 10^−8^). Following colocalization analysis, we observed 259 SNP–cell-type colocalization events across brain cell strata. These events represent counts summed across cell types rather than unique SNP counts; therefore, the same lead SNP could contribute to more than one colocalization event if it colocalized with eQTL signals in multiple brain cell types. Cell type specificity was observed for 17.4–89.2% of colocalized SNPs and 14.6–80.5% of colocalized genes, as measured by the proportion of SNPs or genes detected in a single cell type versus across multiple cell types (Figure 2A). Cross-cell-type analysis of eQTL effect sizes revealed a consistent attenuation of effects for colocalized SNPs in cellular contexts other than their primary cell type of action (Figure 2B), supporting a model of cell type-specific regulation of gene expression by these lipid-associated variants. Furthermore, 77.8% of colocalized genes exhibited cell type-restricted expression patterns (Figure 2C). A representative LocusZoom plot illustrates cell-type-specific eQTL signals at the POMC locus across astrocytes, endothelial cells, excitatory neurons, and other cell types (Figure 2D). Full colocalization results are provided in Table S1, and LDSC genetic correlation estimates between lipid traits and kidney outcomes, including genetic correlation coefficients, standard errors, and *p* values, are provided in Table S5.

Unless otherwise stated, the Results report primary estimates based on the prespecified instrument set before radial or MR-PRESSO outlier removal. Outlier-corrected estimates are presented as sensitivity analyses and are reported in the Supplementary Tables.

### 3.2. Cell-Type- Stratified Causal Effects of Total Cholesterol

Because the summary-level data did not distinguish individual glomerular disease subtypes, all CGN-related associations were interpreted as broad chronic glomerular disease signals rather than subtype-specific mechanisms. Before interpreting the cell-type-stratified MR results, we clarified the effect scale used throughout the Results. For binary renal outcomes, β values represent log-odds effects, whereas ORs represent exponentiated effects. Thus, β = 0 and OR = 1 indicate the null effect. Rounded estimates close to 1.000 in the figures or Supplementary Tables should be interpreted as ORs close to the null value rather than β values equal to 1.000. We distinguished multi-instrument associations from single-instrument findings. Associations supported by two or more SNPs were primarily interpreted using IVW estimates together with available sensitivity analyses, whereas associations supported by only one SNP were reported as Wald-ratio estimates. Single-SNP Wald-ratio findings were considered provisional because heterogeneity and directional pleiotropy could not be formally assessed. Accordingly, CGN-related results were not interpreted as evidence for mechanisms specific to IgA nephropathy, membranous nephropathy, focal segmental glomerulosclerosis, lupus nephritis, or other individual glomerular disease subtypes. CGN was interpreted as a broad chronic glomerular disease phenotype, and the results should not be taken as subtype-specific evidence for a particular histopathological form of glomerulonephritis.

Primary MR analysis using all genome-wide significant variants as IVs established causal associations between TC and hypertensive nephropathy, CKD, and IgA nephropathy. LD Score Regression further corroborated these links.

After correction for multiple testing, total cholesterol showed cell-type-specific associations with several renal outcomes, particularly renal papillary adenocarcinoma, chronic glomerulonephritis, and chronic kidney disease. These associations were observed across multiple brain cell contexts, including endothelial cells, astrocytes, cortical tissue, frontal cortex, excitatory neurons, inhibitory neurons, microglia, oligodendrocyte precursor/committed oligodendrocyte lineage cells, oligodendrocytes, and pericytes. These findings suggest that cholesterol-related genetic regulation may influence renal disease through distributed but cell-type-dependent brain regulatory mechanisms.

The strong oligodendrocyte involvement in TC-associated with broad chronic glomerular disease risk likely reflects the high cholesterol demand of myelination. Disrupted myelin lipid homeostasis may trigger neuroinflammation and subsequently affect renal function through sympathetic outflow pathways. Full colocalization results are provided in Table S1. Conventional MR and csMR results for total cholesterol are provided in Table S3a,b, with sensitivity analyses in Table S4a.

### 3.3. Co-Localization and Causal Effects of Very Low-Density Lipoprotein Cholesterol (VLDL-C)

**Figure 3:**
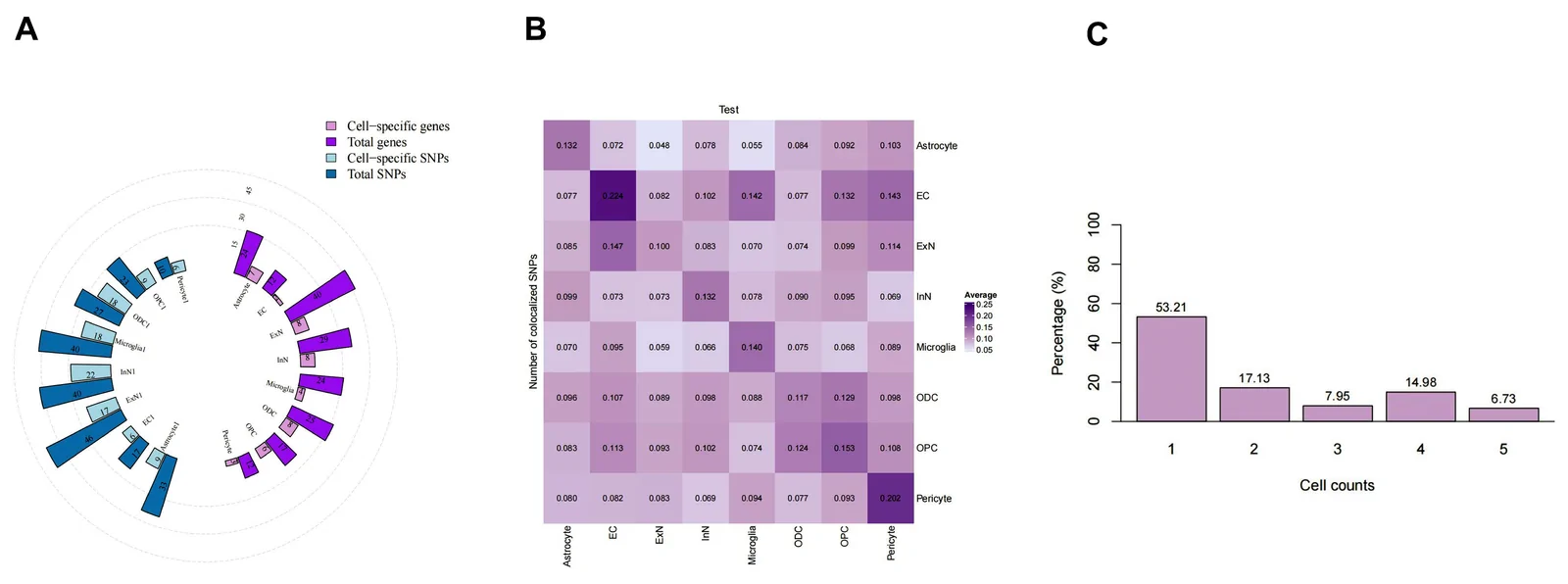
(**A**) Quantitative profiling of colocalization signals, illustrating aggregate and cell type-specific counts of colocalized SNPs and their target genes. (**B**) Comparative assessment of cross-cell-type eQTL effects. (**C**) Cellular tropism of colocalized SNPs: proportional distribution of SNPs detected across variable numbers of cell types, demonstrating predominant single-type-specific colocalization.

GWAS analysis identified 59 near-independent lead SNPs associated with VLDL-C. Colocalization identified 46 SNPs influencing ~40 genes, with 27.2–60.0% of SNPs showing cell-type specificity (Figure 3A–C).

csMR analysis showed that VLDL-C was associated with chronic glomerulonephritis (CGN) in a cell-type-stratified pattern. The pericyte stratum showed an inverse association with CGN (β = −0.688 on the log-odds scale; *p* = 3.23 × 10^−26^), whereas endothelial cells, microglia, and oligodendrocytes showed positive log-odds estimates. These results indicate that the direction of the VLDL-C association differed across brain cell strata rather than showing a uniform effect across all cell types. Full β estimates, ORs, confidence intervals, and *p* values are provided in Table S3c,d.

The inverse pericyte-stratified association should be interpreted as a cell-type-stratified genetic association, not as evidence of a confirmed pericyte-mediated protective mechanism. Pericyte and vascular biology may provide a plausible context for future studies, but the present analysis did not directly assess blood–brain barrier function, VLDL receptor signaling, or renal protection mechanisms. Conventional MR and csMR results for VLDL-C are provided in Table S3c,d, with sensitivity analyses in Table S4b.

### 3.4. Co-Localization and Causal Effects of High-Density Lipoprotein Cholesterol (HDL-C)

**Figure 4:**
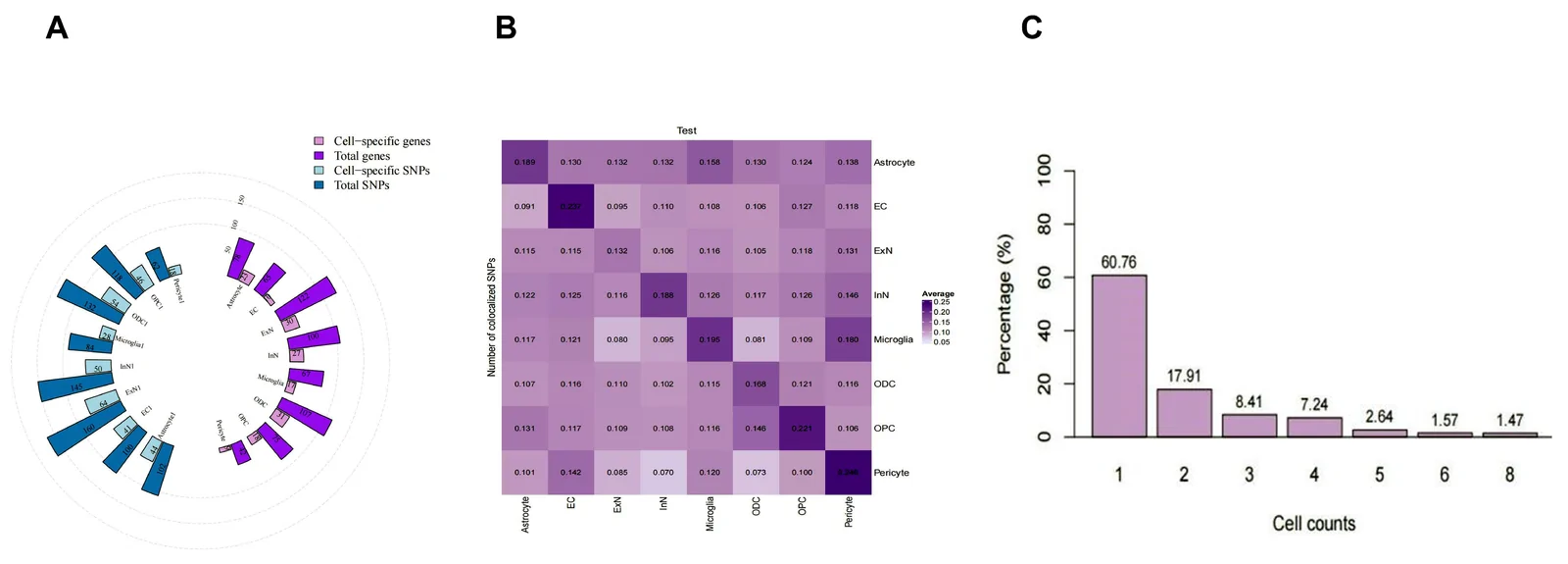
(**A**) Quantitative profiling of colocalization signatures, enumerating aggregate and cell type-resolved counts of colocalized SNPs and their regulated genes. (**B**) Cross-cellular eQTL effect analysis. (**C**) Cellular tropism of colocalized SNPs: proportional distribution stratified by number of exhibiting cell types, demonstrating predominant single-restricted colocalization.

Genome-wide association analysis identified 455 near-independent lead SNPs associated with HDL-C. Colocalization identified 160 SNPs across brain cell types (Figure 4A). Cross-cell-type eQTL effect analysis demonstrated attenuated effects in non-primary cell types (Figure 4B). Cellular tropism analysis showed predominant single-restricted colocalization (Figure 4C).

csMR analysis revealed that HDL-C showed positive log-odds associations with CGN in astrocytes, cortical tissue, and microglia. Representative estimates included astrocytes (β = 0.479; *p* = 2.84 × 10^−16^), cortical tissue (β = 0.329; *p* = 6.33 × 10^−27^), and microglia (β = 0.237; *p* = 1.83 × 10^−6^). Corresponding ORs and full-precision estimates are reported in Table S3e,f.

Biological interpretation. Unlike cholesterol-related lipids, HDL-C acts primarily through glial cells (astrocytes and microglia), suggesting an inflammatory rather than a metabolic mechanism. HDL-C may modulate renal risk by altering glial cytokine secretion and systemic inflammatory tone. Conventional MR and csMR results for HDL-C are provided in Table S3e,f, with sensitivity analyses in Table S4c.

### 3.5. Co-Localization and Causal Effects of Low-Density Lipoprotein Cholesterol (LDL-C)

**Figure 5:**
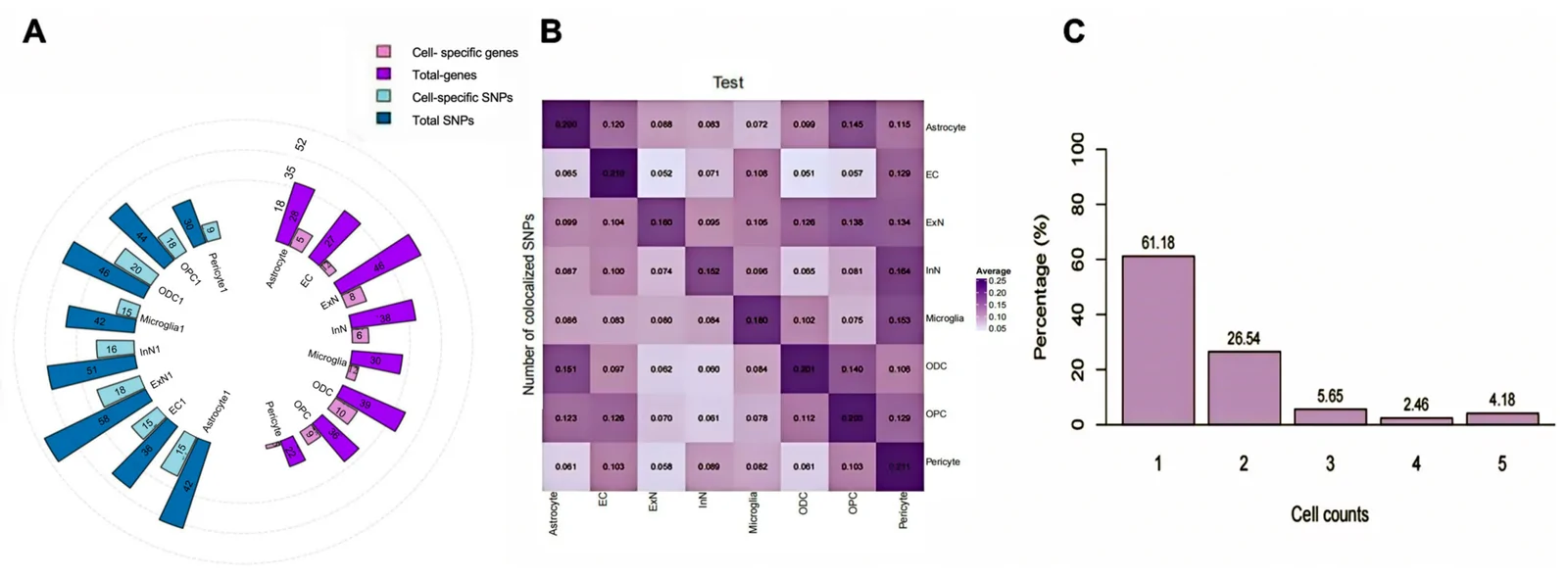
(**A**) Quantitative assessment of colocalization signals enumerating aggregate and cell type-resolved counts of colocalized SNPs and their regulated genes. (**B**) Cross-cellular eQTL effect comparison. (**C**) Cellular distributional bias of colocalized SNPs: proportional representation stratified by number of exhibiting cell types, demonstrating predominant single-cell-type-restricted colocalization.

Genome-wide association analysis identified 194 near-independent lead SNPs associated with LDL-C. Colocalization identified 58 colocalized SNPs (Figure 5A). Cross-cell-type eQTL effect analysis and cellular distributional bias assessment showed predominant cell-type-restricted colocalization (Figure 5B,C).

csMR analysis showed an LDL-C-associated risk pattern like that observed for other cholesterol-related traits. LDL-C showed positive log-odds associations with CGN across several brain cell or tissue strata, including frontal cortex (β = 0.985; *p* = 9.52 × 10^−96^), endothelial cells (β = 1.035; *p* = 1.08 × 10^−113^), and excitatory neurons (β = 0.961; *p* = 2.69 × 10^−132^). These β values are reported on the log-odds scale; corresponding ORs and full-precision estimates are provided in Table S3g,h.

The excitatory neuron-specific LDL signal suggests that LDL-C may activate neuronal pathways—potentially within the paraventricular nucleus—that enhance renal sympathetic output, linking hyperlipidemia to hypertensive renal injury. Conventional MR and csMR results for LDL-C are provided in Table S3g,h, with sensitivity analyses in Table S4d.

### 3.6. Co-Localization and Causal Effects of Triglycerides (TG)

**Figure 6:**
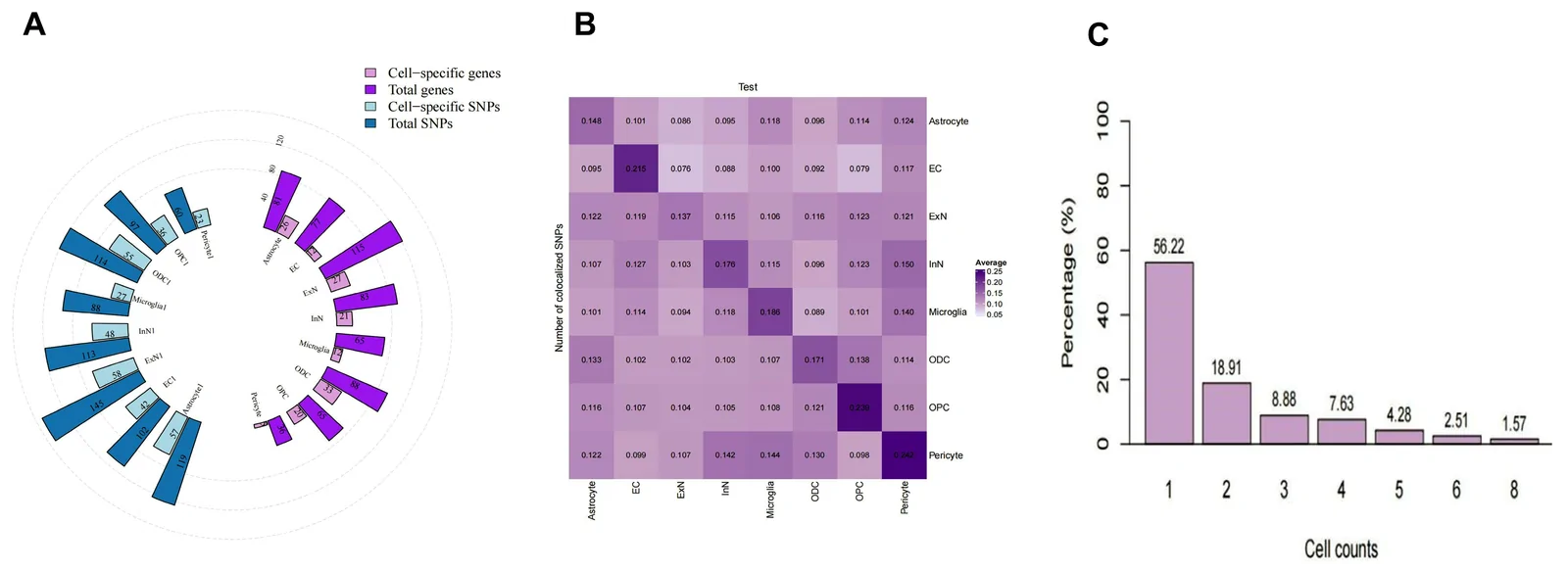
Cell-type-specific colocalization patterns of triglyceride-associated genetic variants in the human brain. (**A**) Numbers of total and cell-type-specific colocalized SNPs and target genes across major brain cell types. (**B**) Cross-cell-type comparison of eQTL effect sizes, showing whether triglyceride-associated variants exert stronger regulatory effects in specific cellular contexts. (**C**) Distribution of colocalized SNPs according to the number of brain cell types in which they were detected.

Genome-wide association analysis identified 392 near-independent lead SNPs associated with TG. Colocalization identified 145 SNPs (Figure 6A). Cross-cell-type eQTL effect comparison and variant regulatory topography analysis demonstrated predominant single-cell-type-restricted colocalization across brain cell strata (Figure 6B,C).

csMR analysis showed that TG elevation increased broad chronic glomerular disease risk through astrocytes (β = 0.264; *p* = 1.75 × 10^−4^) and brain cortex (β = 0.167; *p* = 6.81 × 10^−6^), with more modest effect sizes than cholesterol-related lipids. The cell-type-stratified heatmap for TG is provided in Figure S6 (Supplementary Materials).

The astrocyte-dominant TG pathway, shared with HDL-C, points to a common glial response to lipid surplus. The smaller effect magnitudes suggest TG plays a secondary role to cholesterol in brain-mediated renal injury. Conventional MR and csMR results for triglycerides are provided in Table S3i,j, with sensitivity analyses in Table S4e.

### 3.7. Cell-Type-Specific Effects of Triglycerides on Renal Outcomes

We next examined whether triglyceride-associated genetic variants showed cell-type-specific effects on renal outcomes. In the initial MR analysis, genetically predicted triglyceride levels were associated with chronic kidney disease. Complementary LDSC analysis supported additional associations with kidney cancer excluding renal pelvis, chronic glomerulonephritis, hypertensive nephropathy, chronic kidney disease, and renal failure. Detailed phenotype identifiers and full statistical results are provided in Tables S3i,j, S4e and S5.

After quality control and multiple-testing correction, triglyceride showed cell-type-specific associations with chronic glomerulonephritis. These associations were observed across astrocytes, cortical tissue, frontal cortex, and oligodendrocyte precursor/committed oligodendrocyte lineage cells. This pattern suggests that triglyceride-related genetic regulation may influence chronic glomerulonephritis through multiple brain-cell contexts rather than through a single isolated cell type.These cell-type-specific findings are summarized in Table 3.

The triglyceride-related signals for chronic glomerulonephritis were observed in astrocytes, cortical tissue, frontal cortex, and oligodendrocyte lineage cells. Across these cell contexts, genetically predicted triglyceride elevation was consistently associated with increased disease risk, supporting a convergent but cell-type-dependent role of triglyceride metabolism in chronic glomerulonephritis. Lipid–protein association results used for mediation pathway construction are provided in Table S6, and final mediation analysis results are provided in Table S2.

**Table 3:** Summary of principal cell-type-stratified MR findings and candidate protein-mediated pathways.

Evidence Layer	Lipid and Brain Stratum	Renal Outcome	Protein	Main Biological Interpretation	Key Estimate/Source
Cell-type-stratified MR	Total cholesterol; multiple brain cell/tissue strata	Renal papillary adenocarcinoma; chronic glomerulonephritis; chronic kidney disease	—	Distributed cholesterol-related associations across multiple brain cell/tissue contexts.	Representative results in Results; full estimates in Tables S3a,b and S4a.
Cell-type-stratified MR	VLDL-C; pericytes	Chronic glomerulonephritis	—	Protective association most evident in the pericyte stratum.	β = −0.688; *p* = 3.23 × 10^−26^; Tables S3c,d and S4b.
Cell-type-stratified MR	HDL-C; astrocytes, cortical tissue, and microglia	Chronic glomerulonephritis	—	Positive log-odds associations across glial/cortical strata.	β = 0.479, 0.329, and 0.237; all *p* ≤ 1.83 × 10^−6^; Tables S3e,f and S4c.
Cell-type-stratified MR	LDL-C; frontal cortex, endothelial cells, and excitatory neurons	Chronic glomerulonephritis	—	Positive log-odds associations across neuronal, endothelial, and cortical strata.	β = 0.985, 1.035, and 0.961; all *p* ≤ 9.52 × 10^−96^; Tables S3g,h and S4d.
Cell-type-stratified MR	Triglycerides; astrocytes and cortical tissue	Chronic glomerulonephritis	—	Positive associations with smaller effect sizes than cholesterol-related traits.	β = 0.264 and 0.167; *p* = 1.75 × 10^−4^ and 6.81 × 10^−6^; Tables S3i,j and S4e.
Candidate protein-mediated pathway	Total cholesterol; inhibitory neurons	Chronic glomerulonephritis-related outcome	TIMD4	Opposite-direction statistical mediation signal.	Indirect effect = −0.0186; mediation proportion = −8.83%; *p* = 6.14 × 10^−4^; Table S2.
Candidate protein-mediated pathway	Total cholesterol; oligodendrocyte precursor/committed oligodendrocyte lineage cells	Chronic kidney disease	ICAM4	Candidate protein-mediated pathway; interpreted cautiously.	Indirect effect = −0.0859; mediation proportion = 18.50%; *p* = 0.0231; Table S2.
Candidate protein-mediated pathway	Total cholesterol; endothelial cells	Renal papillary adenocarcinoma	OXT	Exploratory positive statistical mediation signal.	Indirect effect = 0.0899; mediation proportion = 1.54%; *p* = 0.0260; Table S2.
Candidate protein-mediated pathway	LDL-C; cortical tissue	Chronic glomerulonephritis-related outcome	TIMD4	Exploratory positive statistical mediation signal.	Indirect effect = 0.0105; mediation proportion = 1.10%; *p* = 0.0272; Table S2.
Candidate protein-mediated pathway	Total cholesterol; frontal cortex	Chronic glomerulonephritis-related outcome	TIMD4	Exploratory positive statistical mediation signal.	Indirect effect = 0.0212; mediation proportion = 2.12%; *p* = 0.0350; Table S2.
Candidate protein-mediated pathway	Total cholesterol; endothelial cells	Renal papillary adenocarcinoma	CTSF	Opposite-direction or suppressor-like statistical mediation signal.	Indirect effect = −0.6379; mediation proportion = −10.94%; *p* = 0.0440; Table S2.
Candidate protein-mediated pathway	Total cholesterol; endothelial cells	Renal papillary adenocarcinoma	TP53I3	Exploratory positive statistical mediation signal.	Indirect effect = 0.1148; mediation proportion = 1.97%; *p* = 0.0457; Table S2.
Candidate protein-mediated pathway	Total cholesterol; microglia	Kidney and ureteral calculi	SNAP29	Opposite-direction or suppressor-like statistical mediation signal.	Indirect effect = −0.1306; mediation proportion = −16.23%; *p* = 0.0482; Table S2.

This main-text table summarizes the principal findings; complete estimator-level outputs and sensitivity analyses are provided in Tables S1–S7. Abbreviations: MR, Mendelian randomization; VLDL-C, very-low-density lipoprotein cholesterol; HDL-C, high-density lipoprotein cholesterol; LDL-C, low-density lipoprotein cholesterol.

### 3.8. Replicated Plasma Candidate Protein Mediators of Renal Diseases

Our cell-type-specific Mendelian randomization (csMR) analyses identified lipid exposures with cell-type-specific causal effects on renal outcomes. To translate these genetic insights into actionable therapeutic hypotheses, we focused the present study on four renal disease outcomes that exhibited the most robust cell-type-specific effects in our csMR framework, including chronic glomerulonephritis, kidney and ureteral calculi, chronic kidney disease and renal papillary adenocarcinoma. Additional analyses for other outcomes are provided in the Supplementary Tables and Figures.

**Figure 7:**
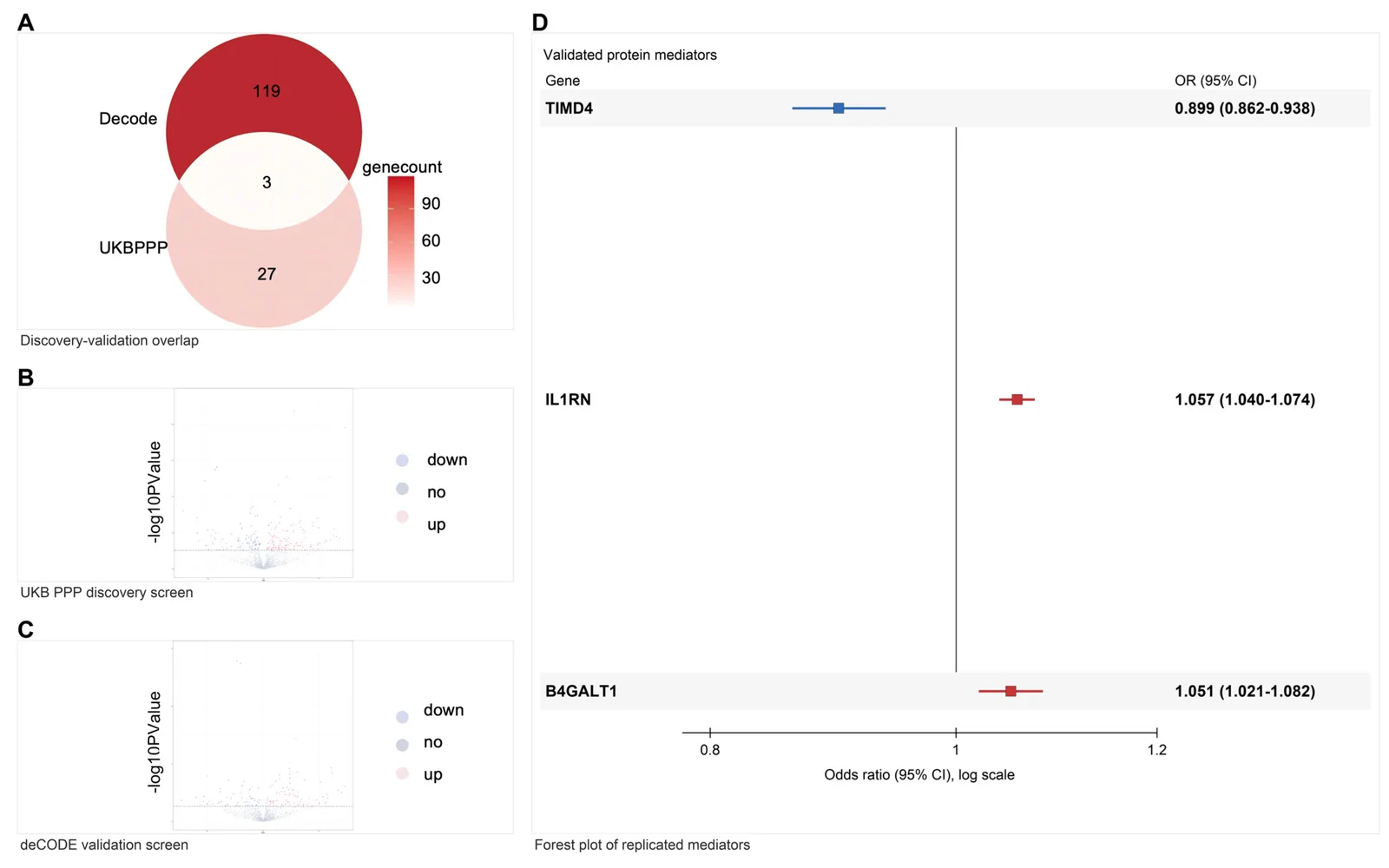
Externally supported plasma protein associations with chronic kidney disease. (**A**) Discovery–validation overlap of plasma protein associations with chronic kidney disease between the UK Biobank Pharma Proteomics Project (UKB–PPP) and deCODE cohorts. (**B**) Protein–phenotype association screen in the UKB-PPP discovery cohort. (**C**) Protein–phenotype association screen in the deCODE validation cohort. (**D**) Directionally consistent protein associations, highlighting TIMD4, IL1RN, and B4GALT1 as candidate protein signals related to immune and extracellular-matrix biological themes. These panels summarize association-based discovery and validation rather than systematic pQTL–MR or protein–outcome colocalization.

**Figure 8:**
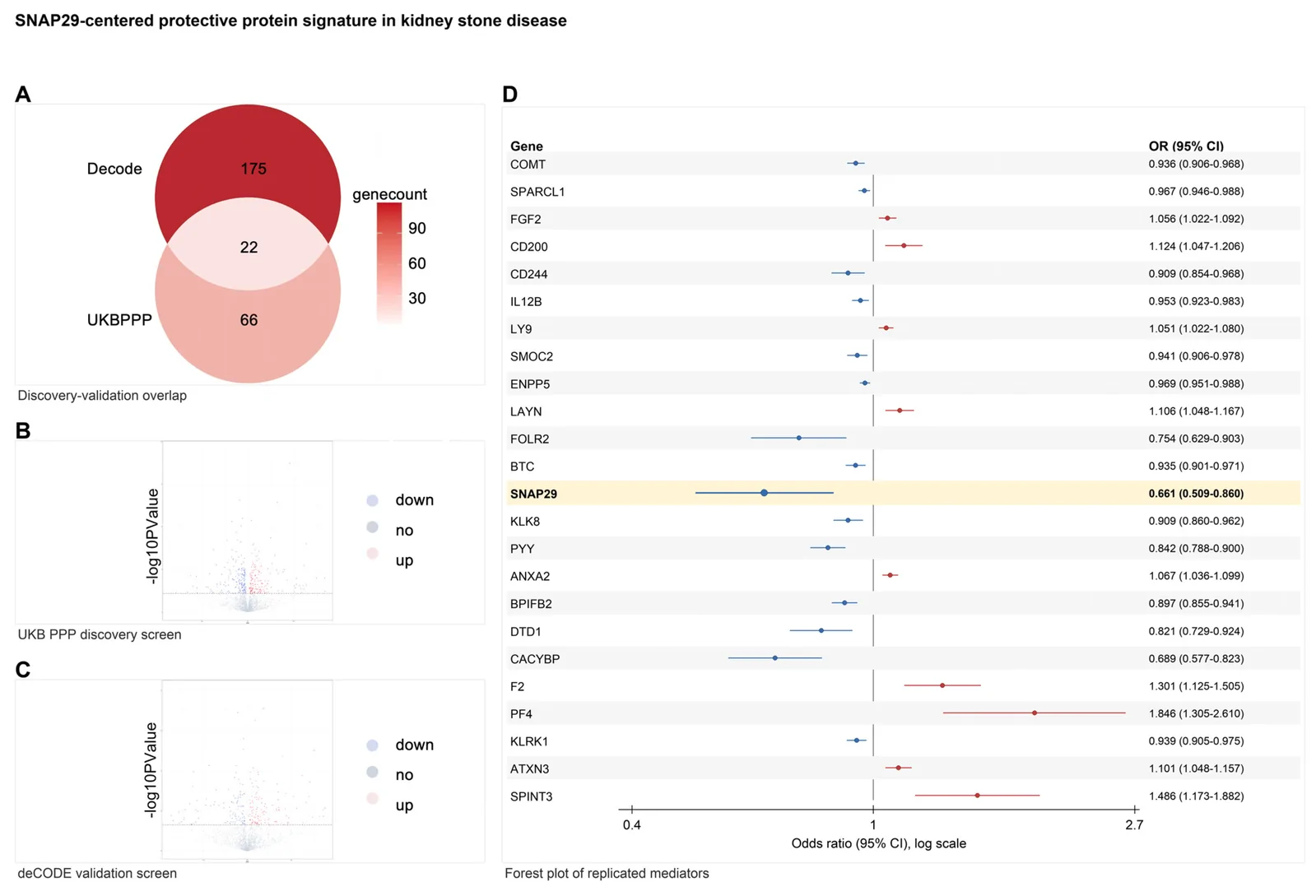
Functional interpretation of replicated plasma protein associations with kidney and ureteral calculi. (**A**) Overlap of protein associations between the UKB-PPP discovery cohort and the deCODE validation cohort. (**B**) STRING−based protein–protein interaction network of replicated proteins. (**C**) Functional annotation of replicated proteins showing enriched biological themes. (**D**) Directionally consistent replicated protein associations. These analyses were used to support biological interpretation and should be considered exploratory.

**Figure 9:**
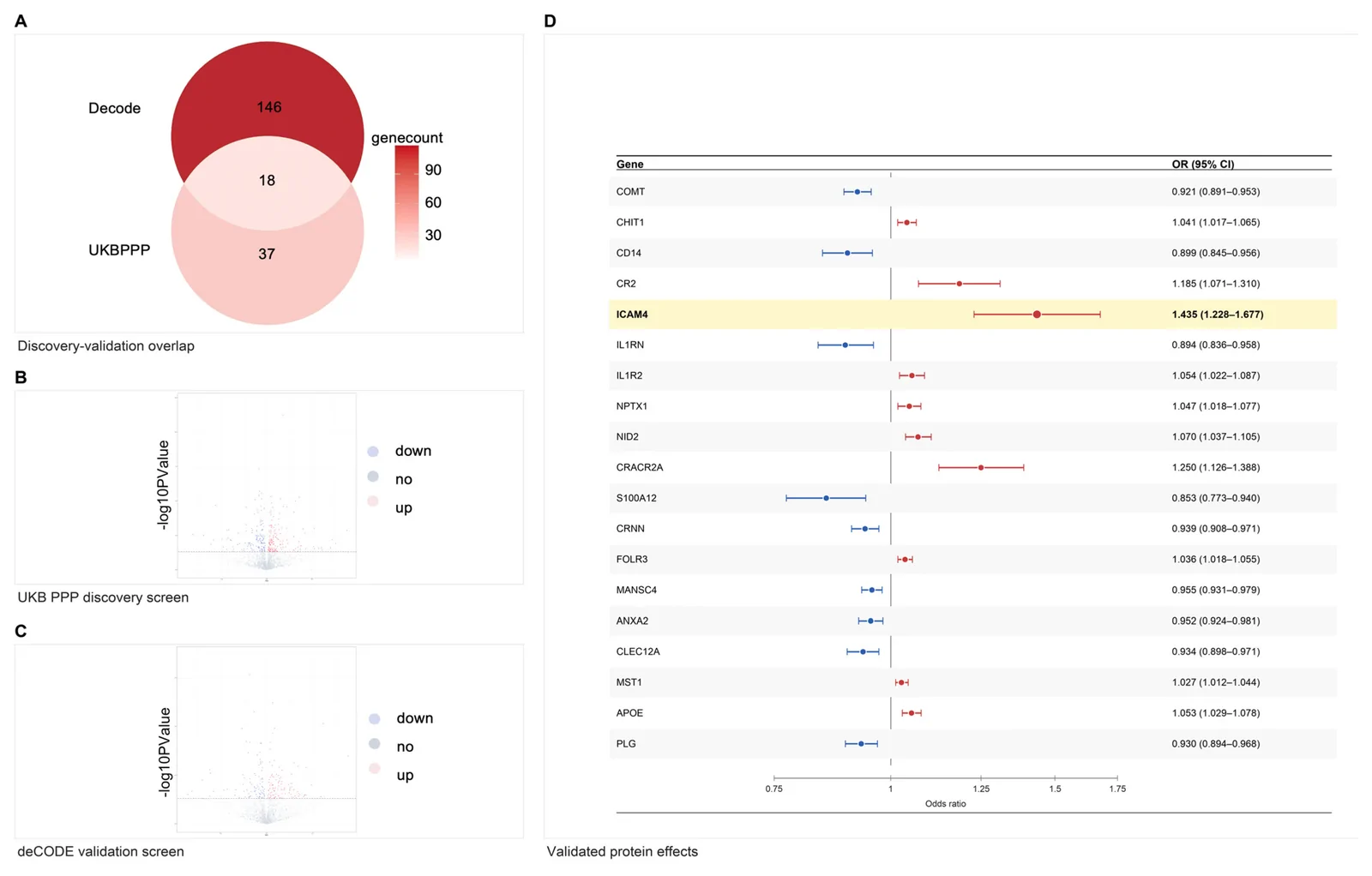
Functional interpretation of replicated plasma protein associations with chronic kidney disease. (**A**) Overlap of protein associations between the UKB-PPP discovery cohort and the deCODE validation cohort. (**B**) STRING−based protein–protein interaction network of replicated proteins. (**C**) Functional annotation of replicated proteins highlighting immune, inflammatory, adhesion, or vascular-related biological themes. (**D**) Directionally consistent replicated protein associations. These findings represent exploratory protein-association and annotation signals rather than confirmed causal protein−mediated pathways.

**Figure 10:**
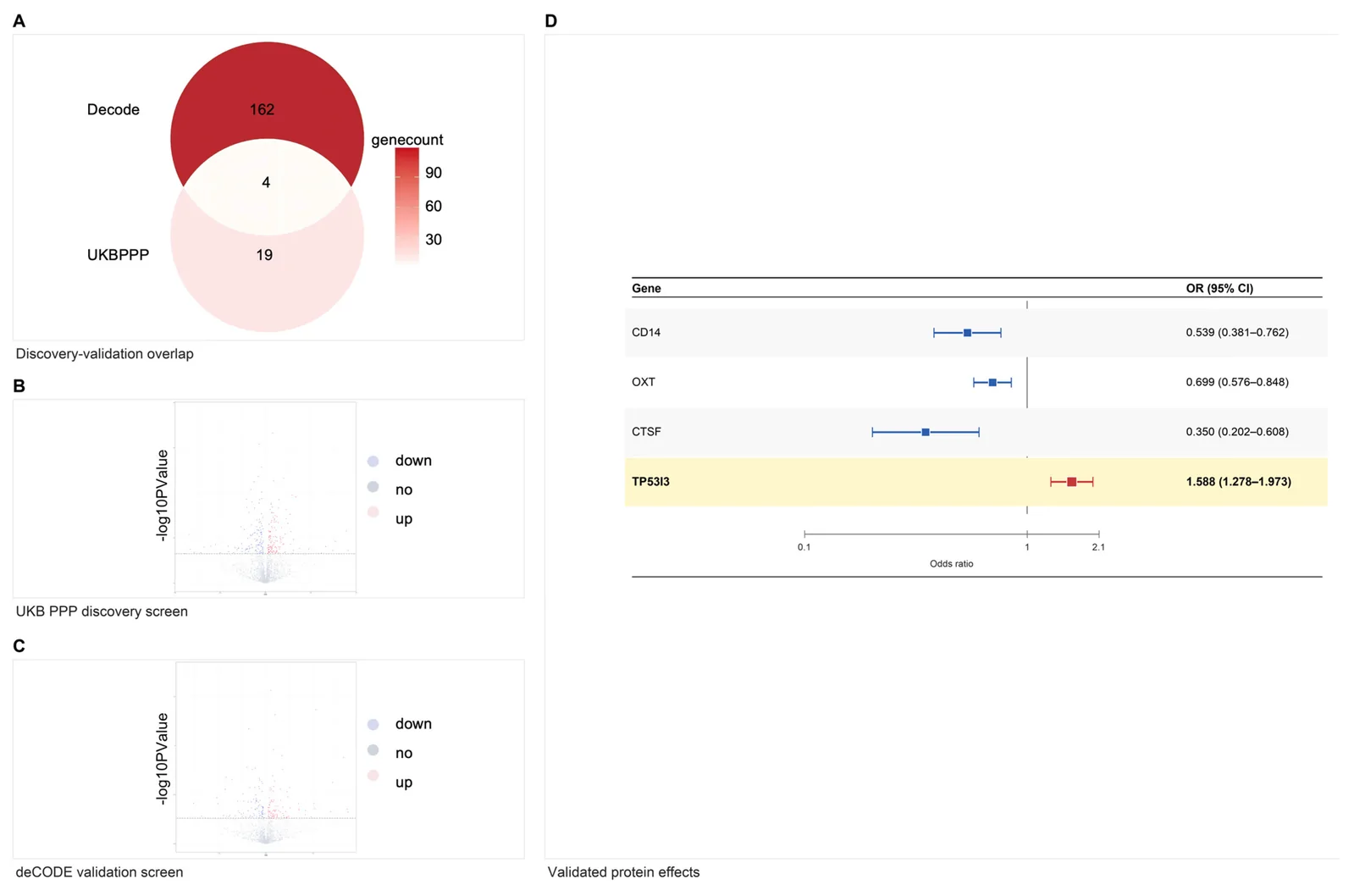
Externally supported plasma protein associations with renal papillary adenocarcinoma. (**A**) Discovery–validation overlap of plasma protein associations with renal papillary adenocarcinoma between the UKB−PPP and deCODE cohorts. (**B**) Protein–phenotype association screen in the UKB−PPP discovery cohort. (**C**) Protein–phenotype association screen in the deCODE validation cohort. (**D**) Directionally consistent protein associations, highlighting CD14, OXT, CTSF, and TP53I3 as exploratory candidate protein signals related to immune, lysosomal, neuroendocrine, and tumor−stress biological themes. These panels summarize association-based discovery and validation rather than systematic pQTL−MR or protein–outcome colocalization.

Before interpreting Figure 7, Figure 8, Figure 9 and Figure 10, we clarified that these figures summarize protein–disease association discovery in UKB-PPP followed by external validation in deCODE. They do not represent systematic pQTL-MR or protein–outcome colocalization analyses. Therefore, the proteins shown in these figures were interpreted as externally supported candidate protein associations rather than confirmed causal protein mediators. To address the biological interpretability of these replicated protein signals, we further summarized the common proteins using STRING-based protein–protein interaction analysis and functional annotation. These analyses were used to identify exploratory functional themes rather than to infer causal protein-mediated mechanisms.

For chronic kidney disease, proteome-wide discovery in the UK Biobank Pharma Proteomics Project (UKB-PPP) cohort followed by external validation in deCODE identified three replicated plasma protein mediators: TIMD4, IL1RN, and B4GALT1. TIMD4 showed a protective association, whereas IL1RN and B4GALT1 showed risk-increasing associations. These proteins point to biologically coherent pathways involving immune regulation, inflammatory signaling, and extracellular-matrix remodeling. Full effect estimates and robustness analyses are provided in Figure 7.

These externally supported protein associations suggest an exploratory immune–inflammatory and extracellular-matrix-related protein signature in chronic kidney disease. TIMD4 may reflect macrophage-related immune regulation, IL1RN may point to interleukin-1-mediated inflammatory control, and B4GALT1 may be related to glycosylation-dependent extracellular-matrix or tissue-remodeling processes. These interpretations are hypothesis-generating and do not establish experimentally confirmed protein-mediated mechanisms.

For kidney and ureteral calculi, proteome-wide discovery in the UKB-PPP cohort followed by deCODE validation identified 22 replicated plasma proteins. Among these, SNAP29 showed a prominent protective association. Given its role in vesicle trafficking and membrane-fusion processes, SNAP29 may reflect a protective trafficking-related protein signature in stone-related renal pathology. Detailed effect estimates and robustness analyses are provided in Figure 8. STRING-based protein–protein interaction and functional annotation analysis of the replicated proteins further suggested that these signals clustered around vesicle trafficking, membrane-fusion, autophagy-related, and immune-related biological themes. These findings provide a functional context for the SNAP29-centered signal but remain exploratory.

SNAP29 is involved in SNARE-mediated vesicle trafficking, membrane fusion, and autophagy-related processes. In our analysis, SNAP29 showed an externally supported association with kidney and ureteral calculi. This signal may provide a hypothesis-generating link to intracellular trafficking or autophagy-related biology, but it should not be interpreted as evidence that SNAP29 biologically mediates stone-related renal pathology.

For chronic kidney disease, proteome-wide discovery in the UKB-PPP cohort followed by deCODE validation identified 18 replicated plasma proteins. ICAM4 showed a prominent risk-increasing association, suggesting the involvement of cell-adhesion and inflammatory pathways in chronic kidney disease. Together with other replicated immune-related proteins, this result supports a coordinated inflammatory and vascular-adhesion protein signature rather than a single isolated biomarker. Full statistical details are provided in Figure 9. Functional annotation of the replicated proteins supported biological themes related to cell adhesion, inflammatory signaling, and vascular or immune regulation. These findings suggest that the CKD-associated plasma protein signature may reflect coordinated inflammatory and adhesion-related processes rather than a single isolated biomarker.

ICAM4 showed an externally supported risk-increasing protein association with chronic kidney disease. As a member of the intercellular adhesion molecule family, ICAM4 may provide a hypothesis-generating signal related to cell-adhesion or inflammatory biology. However, this association should not be interpreted as evidence that ICAM4 is a confirmed mediator or therapeutic target in chronic kidney disease.

For renal papillary adenocarcinoma, proteome-wide discovery in the UKB-PPP cohort identified 23 disease-associated plasma proteins, of which four were replicated in the independent deCODE cohort. These externally supported protein associations suggest exploratory immune, neuroendocrine, lysosomal, and tumor-stress-related biological themes in renal papillary adenocarcinoma (Figure 10A–C).

Among the replicated proteins, CD14, OXT, CTSF, and TP53I3 showed directionally consistent associations. OXT and CTSF were inversely associated with renal papillary adenocarcinoma, whereas TP53I3 showed a risk-increasing association. These proteins suggest several biologically interpretable processes, including immune signaling, neuroendocrine regulation, lysosomal protease activity, and p53-related oxidative-stress responses. Detailed ORs and 95% CIs are shown in Figure 10D.

The replicated proteins associated with renal papillary adenocarcinoma point to several biologically interpretable processes. CD14 suggests innate immune activation, OXT may reflect neuroendocrine regulation, CTSF is related to lysosomal protease activity, and TP53I3 is involved in p53-related oxidative-stress responses. These findings generate hypotheses that immune, lysosomal, neuroendocrine, and tumor-stress-related processes may be relevant to the proteomic signature.

### 3.9. Exploratory Proteomic Mediation Signals

After identifying externally supported protein–disease associations in Figure 7, Figure 8, Figure 9 and Figure 10, we performed a separate exploratory statistical mediation analysis to evaluate whether these candidate protein signals may statistically account for part of the lipid–brain-cell-stratum associations with renal outcomes. The csMR analysis identified 54 lipid–protein associations suggesting that lipid-associated genetic signals annotated to brain cell strata may be statistically linked to circulating protein levels. These associations involved several key proteins, including TIMD4, ICAM4, OXT, CTSF, TP53I3, and SNAP29, across frontal cortex, endothelial cells, microglia, inhibitory neurons, oligodendrocyte lineage cells, and cortical tissue.

The prioritized exploratory mediation analysis identified eight candidate statistical mediation signals involving six proteins: TIMD4, ICAM4, OXT, CTSF, TP53I3, and SNAP29 (Table S2). TIMD4 appeared in three candidate pathways linking cholesterol-related signals in inhibitory neurons, cortical tissue, or frontal cortex to chronic glomerulonephritis-related outcomes. ICAM4 was included in a candidate statistical mediation signal linking a total cholesterol-related oligodendrocyte-lineage stratum with chronic kidney disease, with an estimated mediation proportion of approximately 18.5%. SNAP29 was associated with a microglia-related pathway for kidney and ureteral calculi. For renal papillary adenocarcinoma, OXT, CTSF, and TP53I3 were prioritized in endothelial-cell-related total cholesterol pathways.

Several indirect effects were negative or opposite in direction to the corresponding total or direct effects, including TIMD4, CTSF, and SNAP29. These findings were therefore interpreted as opposite-direction or suppressor-like statistical mediation signals rather than definitive evidence of biological mediation. Overall, the candidate proteins point to immune regulation, cell adhesion, vesicle trafficking, lysosomal activity, neuroendocrine signaling, and tumor-associated stress responses as plausible biological themes requiring further validation. These findings should be interpreted as exploratory statistical mediation signals rather than biologically confirmed protein-mediated pathways. Systematic protein–outcome colocalization and multivariable MR were not performed for all candidate proteins, and several indirect effects showed opposite or suppressor-like directions.

## 4. Discussion

### 4.1. Cellular Heterogeneity: Resolving the Resolution Crisis in Genetic Causal Inference

This study applies an established cell-type-stratified Mendelian randomization (csMR) framework to investigate lipid-related brain cell strata associated with kidney disease. Traditional tissue-level analyses may obscure cell-type-specific regulatory signals by averaging genetic regulatory effects across diverse cell populations. By integrating brain single-cell eQTL atlases with lipid and kidney GWAS, our analysis suggests that lipid-associated genetic signals can be mapped to specific brain cell strata that show MR-supported associations with renal outcomes. These findings should be interpreted as cell-type-resolved genetic evidence rather than proof that lipid-induced kidney disease physically originates in the brain.

Genetically predicted lipid effects linked to brain cell-type-specific eQTL variants should not be interpreted as evidence that the corresponding biological process physically originates in the brain. Rather, these findings indicate that lipid-associated genetic signals can be annotated to specific brain cell strata and are associated with renal outcomes under MR assumptions.

### 4.2. Glial Cell-Mediated Mechanisms in Lipid Homeostasis and Renal Pathophysiology

Our two-sample MR analyses suggest that oligodendrocyte lineage cells and pericytes may contribute to lipid-related genetic effects on renal outcomes, including chronic glomerulonephritis and kidney and ureteral calculi [37]. These findings are consistent with previous MR studies linking lipid profiles to renal disease risk. In particular, prior evidence suggests that LDL cholesterol and apolipoprotein B may adversely affect renal function, whereas HDL cholesterol may have protective associations [38,39]. Together, these results support the hypothesis that lipid metabolism contributes to renal pathology through both systemic and brain-cell-specific regulatory mechanisms.

#### 4.2.1. The Biological Logic of the Brain–Kidney Axis

The conceptual framework of the “brain–kidney axis” provides a useful biological context for interpreting our findings, but it should be regarded here as a hypothesis-generating framework rather than a mechanism directly tested in this study. Prior studies suggest that oligodendrocytes have important roles in lipid-rich myelin biology, that neuron–glia metabolic coupling may participate in systemic metabolic regulation, that hypothalamic circuits such as the paraventricular nucleus may influence renal neural control, and that neurovascular interfaces may respond to lipid-related stress [40,41,42,43,44,45,46]. These studies provide biological plausibility for the cell-type-stratified associations observed in our analysis. However, our csMR analyses did not directly measure oligodendrocyte lipid sensing, PVN activity, sympathetic outflow, blood–brain barrier transport, neurovascular signaling, or exosome-mediated brain-to-kidney communication.

Several possible routes may be considered for future experimental investigation. Autonomic and neuroendocrine pathways could plausibly connect central metabolic regulation with renal hemodynamics and sodium handling, while inflammatory, neurovascular, or extracellular-vesicle-related processes may represent additional candidate routes of inter-organ signaling. Importantly, these routes were not directly assessed in the present study, and our results should not be interpreted as evidence that any specific brain-to-kidney communication mechanism has been established.

#### 4.2.2. Oligodendrocyte-Related Lipid Signals as a Hypothesis-Generating Finding

Our analyses identified oligodendrocyte-related lipid signals associated with chronic glomerular disease outcomes. For example, HDL-C showed a strong association in oligodendrocyte-stratified analyses (IVW β = 1.025 on the log-odds scale, 95% CI 0.964–1.085; *p* = 9.76 × 10^−243^). Because oligodendrocytes have high lipid and cholesterol demands for myelin formation, this finding is biologically plausible considering prior literature on myelin lipid metabolism and lipoprotein receptor expression in myelinating oligodendrocytes [40,41]. However, the present study did not directly assess oligodendrocyte lipid uptake, myelination dynamics, or renal injury mechanisms. Therefore, the oligodendrocyte-related findings should be interpreted as genetic association signals that prioritize this cell lineage for future functional studies, rather than as evidence of a confirmed oligodendrocyte-mediated mechanism.

### 4.3. Neuronal-Stratum Associations as Hypothesis-Generating Signals

This study identified neuron-stratified genetic associations between lipid traits and renal outcomes, but it does not directly establish specialized neuronal functions or neuronal mediation of kidney disease. LDL-C associations with the broad CGN phenotype differed across neuronal strata. Excitatory neurons showed a positive association on the log-odds scale (β = 0.961, 95% CI 0.936–0.987; *p* < 1 × 10^−300^), whereas inhibitory neurons showed an inverse association (β = −0.464, 95% CI −0.560 to −0.368; *p* = 4.94 × 10^−5^). The extremely small *p* value for the excitatory-neuron estimate reached computational underflow and is therefore reported using lower-bound notation.

The following observations support a model of neuronal “bidirectional switching: Pathological Instigation: Activation of excitatory neurons, likely within the paraventricular nucleus (PVN), enhances sympathetic outflow to the kidney which provides biological context for future studies but was not directly tested here [43]. Protective Reflexes: Inhibitory neurons may trigger vagal-anti-inflammatory reflexes. Stimulation of the dorsal motor nucleus (DMV) has been shown to lower albumin excretion and interstitial fibrosis [35]. We further identify excitatory neurons as novel regulators of lipid-bone crosstalk (HDL reducing nephrolithiasis risk, *β* = 0.805), aligning with canonical AgRP circuitry that coordinates bone-lipid homeostasis via NPY pathways [47].

### 4.4. Endothelial- and Pericyte-Stratified Vascular Signals

Endothelial-cell- and pericyte-stratified analyses highlighted candidate vascular or perivascular cell strata associated with lipid-related renal outcomes. TC in endothelial cell-stratified analyses showed a strong positive association with renal papillary adenocarcinoma risk (IVW β = 5.832 on the log-odds scale, 95% CI 3.096–8.567; *p* = 2.93 × 10^−5^). Given the large effect estimate and wide confidence interval, this association should be interpreted cautiously and requires further validation. Pericyte-stratified analyses showed a VLDL’s renal protection (*β* = −0.688; *p* = 3.23 × 10^−26^).

Endothelial cells, astrocytes, and pericytes are key components of the neurovascular unit and blood–brain barrier [44]. Prior studies also suggest that lipid-related stress may influence BBB transport and astrocyte lipid responses, whereas pericyte biology is relevant to vascular integrity and kidney function [45,46]. These biological functions provide a plausible context for interpreting the endothelial- and pericyte-stratified associations observed in our study. However, we did not directly assess blood–brain barrier permeability, pericyte activation, cytokine release, or renal inflammatory responses [48]. Therefore, these findings should be interpreted as vascular-cell-stratified genetic associations that generate hypotheses for future mechanistic studies.

### 4.5. Biological Interpretation and Translational Relevance of Candidate Protein Mediators

Our proteomic and mediation analyses identified several candidate protein-associated statistical mediation signals that may link lipid-related brain-cell-stratified genetic associations to renal outcomes. These proteins should not be interpreted as experimentally validated causal mechanisms, but rather as genetically supported and proteomic replicated signals that generate biologically plausible hypotheses for future studies.

For chronic kidney disease, TIMD4, IL1RN, and B4GALT1 suggest an immune–inflammatory and tissue-remodeling signature. TIMD4 is commonly linked to macrophage-related immune regulation and apoptotic-cell clearance, which may be relevant to chronic renal inflammation and repair [49,50]. IL1RN encodes the interleukin-1 receptor antagonist and therefore points to IL-1-mediated inflammatory control, a pathway closely related to systemic inflammation and kidney injury [51]. B4GALT1 is involved in glycosylation and may influence extracellular-matrix organization or tissue-remodeling processes. Together, these proteins suggest that chronic kidney disease in our framework may be connected to immune regulation, inflammatory signaling, and extracellular-matrix remodeling [52].

SNAP29 emerged as a protective protein signal in kidney and ureteral calculi. SNAP29 is a SNARE-associated protein involved in vesicle trafficking, membrane fusion, and autophagy-related processes [53]. Because autophagy and intracellular trafficking are important for cellular stress responses in kidney disease, the SNAP29 signal may indicate a protective trafficking-related or autophagy-related pathway in stone-associated renal pathology [54]. However, this interpretation remains hypothesis-generating and requires experimental validation.

ICAM4 showed a risk-increasing association with chronic kidney disease. As a member of the intercellular adhesion molecule family, ICAM4 may reflect cell-adhesion-related inflammatory processes [55,56]. Although most renal literature focuses on ICAM1 and VCAM1 in leukocyte–endothelial interaction, our ICAM4 finding suggests that adhesion-related mechanisms may deserve broader investigation in chronic kidney disease. We therefore interpret ICAM4 as a candidate marker of inflammatory and vascular-adhesion biology rather than as an immediately actionable candidate pathway for future validation.

For renal papillary adenocarcinoma, the replicated proteins CD14, OXT, CTSF, and TP53I3 point to immune, neuroendocrine, lysosomal, and tumor-stress pathways. CD14 is a marker of innate immune activation and has been implicated in renal cancer progression and inflammatory tumor microenvironments [57]. OXT may reflect neuroendocrine regulation, although its role in renal tumor biology remains insufficiently defined [58,59]. CTSF belongs to the cathepsin family and may be related to lysosomal protease activity and tumor-associated matrix remodeling [60]. TP53I3 is a p53-inducible gene associated with oxidative-stress responses, which provides a plausible link to tumor-related stress biology [61]. These observations provide a literature-supported basis for prioritizing these proteins in future functional studies.

The STRING-based interaction and functional annotation analyses provided an additional layer of biological interpretation by showing that several replicated proteins clustered into immune, inflammatory, adhesion, vesicle-trafficking, or extracellular-matrix-related themes. However, these annotations are hypothesis-generating and do not establish direct mechanistic pathways.

Overall, these candidate proteins are consistent with a putative lipid–brain cell–plasma protein–kidney association model. However, they should be interpreted as exploratory statistical mediation signals rather than confirmed biological mediators, because systematic protein–outcome colocalization and multivariable MR were not performed for all candidate proteins, and several indirect effects showed opposite or suppressor-like directions.

### 4.6. Limitations and Future Perspectives

Potential sample overlap and winner’s-curse bias represent additional limitations of this study. Some lipid GWAS, renal-outcome GWAS, and UK Biobank-derived proteomic datasets may share participants, and individual-level data were unavailable to directly remove overlapping samples. Moreover, selecting instruments and estimating exposure effects from the same GWAS may introduce winner’s-curse bias. These issues may be particularly relevant for cell-type-stratified analyses with small instrument sets. We therefore prioritized strong instruments, used external proteomic validation in deCODE where possible, and interpreted small-instrument or potentially overlapping findings as hypothesis-generating rather than definitive causal evidence.

The mediation analysis also has several limitations. First, UKB-PPP and some lipid GWAS may contain overlapping UK Biobank participants, and individual-level data were unavailable to directly remove overlapping samples. Second, the Difference Method relies on assumptions regarding effect-scale compatibility and approximate independence between total and direct effect estimates. Third, protein–outcome colocalization was not systematically performed for all candidate proteins, so shared causal variants between protein and kidney outcome associations could not be confirmed in every pathway. Fourth, because 2923 proteins and multiple lipid–cell–kidney combinations were screened, mediation *p* values around 0.02–0.05 should be regarded as exploratory. Therefore, the candidate protein-mediated pathways require further validation in independent datasets and experimental systems. In addition, Figure 7, Figure 8, Figure 9 and Figure 10 should be interpreted as protein–disease association discovery and validation results, not as evidence from systematic pQTL-MR or protein–outcome colocalization. Therefore, the candidate proteins shown in these figures were used for prioritization and hypothesis generation rather than causal confirmation. Therefore the mediation results should be used for prioritization rather than causal confirmation of protein-mediated biological pathways.

While this study provides robust causal evidence through csMR, several systematic limitations remain and should be the focus of future research. Although we used PhenoScanner filtering, LD clumping, harmonization, and multiple pleiotropy diagnostics to improve instrument validity, these procedures cannot definitively prove the MR independence and exclusion-restriction assumptions. In addition, outlier removal may influence causal estimates if used as a selection procedure. We therefore treated radial MR and MR-PRESSO outlier-corrected results as sensitivity analyses rather than as replacements for the primary estimates, and we interpreted associations according to their consistency across primary and robust MR methods.

#### 4.6.1. Heterogeneity and Ancestry

Some cell-type-specific associations were supported by only one genetic instrument. These single-SNP results were estimated using the Wald-ratio method and could not be evaluated using standard heterogeneity or horizontal pleiotropy tests. Therefore, such findings should be regarded as provisional and hypothesis-generating, requiring validation in larger GWAS, additional cell-type-specific eQTL datasets, or experimental models.

A primary limitation stems from ancestry bias, as the GWAS and UK Biobank data utilized are predominantly of European ancestry. The transferability of Polygenic Risk Scores (PRS) for the brain–kidney axis across Asian or African populations remains to be validated. Genetic architecture varies significantly, and ethnic-specific eQTL atlases are required to refine these causal maps.

#### 4.6.2. Cis- vs. Trans-Regulatory Architecture

Our current framework primarily utilizes cis-eQTLs to ensure high precision and minimal pleiotropy. However, we must acknowledge the critical role of trans-eQTLs and distal enhancers in cross-organ communication. Such cross-organ regulatory relationships often involves long-range chromatin interactions and distal regulatory elements that are not yet fully captured in our model. Future iterations of csMR should integrate trans-regulatory data to provide a more holistic view of the genetic landscape.

#### 4.6.3. Static Cross-Sectional Data vs. Dynamic Progression

The omics data utilized are largely static “snapshots.” Hypertension and renal disease are progressive, involving a transition from early functional hyperfiltration to chronic organ fibrosis. Static eQTLs and proteomic data may not fully capture the temporal dynamics of these pathological processes. Longitudinal multi-omics studies will be essential to map the temporal sequence of molecular events.

#### 4.6.4. Necessity for Functional Validation

Although csMR provides strong statistical support for the inferred relationships, it remains a statistical inference. Biological confirmation is paramount. We propose that subsequent studies utilize Organ-on-a-chip technology—specifically brain–kidney chips—to observe real-time signaling between ODCs/pericytes and renal tubule cells. Furthermore, Human Kidney Organoids can be used to validate the effect of ICAM4 or SNAP29 on tubular architecture and fibrosis in a controlled human genetic environment.

## 5. Conclusions

In this study, we have applied a cell-type-stratified Mendelian randomization (csMR) framework, integrating large-scale genetics with brain single-cell expression quantitative trait locus (eQTL) atlases. Our findings systematically map a “lipid → brain cell → plasma protein → kidney disease” association framework. These findings provide genetic and proteomic association evidence supporting a hypothesis-generating cross-organ perspective on metabolic renal disease. They do not establish direct brain-to-kidney communication or experimentally confirmed mechanisms.

### 5.1. Principal Discoveries

Cell-Type-Stratified Associations Between Lipids and Kidney Disease: Our findings suggest that lipid-associated genetic effects on kidney disease differ across brain cell and tissue strata. Oligodendrocyte- and astrocyte-related analyses showed prominent associations for cholesterol-related traits, whereas neuronal strata showed directionally distinct LDL-related associations. These results prioritize specific brain cell populations for future functional studies but do not establish direct cellular mechanisms.

Chronic Glomerulonephritis as a Recurrent Renal Outcome: CGN appeared as a recurrent renal outcome across several lipid traits and brain cell strata. However, CGN was analyzed as a broad chronic glomerular disease phenotype, and the available summary-level data did not allow us to distinguish specific histopathological subtypes, such as IgA nephropathy, membranous nephropathy, focal segmental glomerulosclerosis, or lupus nephritis. Therefore, CGN-related signals should be interpreted as associations with a heterogeneous chronic glomerular disease category rather than subtype-specific mechanisms.

Exploratory Plasma Protein Mediation Signals: By integrating plasma proteomic GWAS, we prioritized several candidate plasma proteins, including SNAP29, ICAM4, OXT, and TP53I3, that may statistically link lipid-related brain-cell strata with renal outcomes. For example, ICAM4 was included in an exploratory statistical mediation signal associated with chronic kidney disease, with an estimated mediation proportion of approximately 18.5%. These findings should be interpreted as exploratory statistical mediation signals rather than confirmed biological mechanisms.

Methodological Value of Cell-Type-Resolved Inference: The csMR framework overcomes the resolution constraints of traditional tissue-homogenization analyses. By leveraging single-cell resolution, cell-type-specific eQTL information, this approach complements conventional tissue-level MR and helps generate more refined hypotheses about multi-organ regulatory networks.

### 5.2. Significance and Clinical Relevance

Cross-Organ Perspective on Metabolic Kidney Disease: This research provides empirical evidence that broad chronic glomerular disease outcomes, including the CGN phenotype analyzed here, may be studied from a systemic genetic-association perspective that includes lipid-associated brain-cell-stratified signals that include lipid-associated brain-cell signals. This perspective offers a holistic biological framework for understanding the systemic contributors to metabolic renal disease phenotypes.

Candidate Signals for Future Validation: candidate genetic and protein-associated signals identified in this study, including oligodendrocyte-related cholesterol signals, pericyte-related VLDL associations, and ICAM4-related protein signals, may inform future functional validation studies. However, they should not yet be regarded as validated therapeutic targets.

Advancing Single-Cell Causal Medicine: The established csMR framework may be useful for investigating other cross-organ regulatory questions, provided that appropriate cell-type-specific eQTL datasets and well-powered GWAS resources are available. This study provides a robust tool for the post-GWAS era, facilitating the transition from genetic association to cellular-level functional mechanism.

In summary, this work delivers a high-resolution map of the brain–kidney axis and provides candidate cell-type-aware pathways for future mechanistic and translational studies in renal disease. Our findings should be interpreted as hypothesis-generating genetic and proteomic evidence that may inform future experimental studies and cell-type-aware therapeutic exploration in metabolic kidney disease.

## Figures and Tables

**Table 1:** Summary of single-cell and bulk tissue eQTL datasets used in this study.

Feature	Single-Cell eQTL (sc-eQTL)	Bulk Tissue eQTL (GTEx v8)
**Source**	Bryois et al. (2020) [7]	GTEx Consortium
**Tissue/Region**	Prefrontal & Temporal Cortex	Cortex & Frontal Cortex (BA9)
**Ancestry**	European (N = 192)	European (N ≈ 200–800 *)
**Cell Types/Units**	8 Major Cell Types (ExN, InN, etc.)	Homogenized Tissue
**SNP Count**	5.3 million	10.4 million
**Gene Count**	14,595	~20,000
Platform	Droplet-based scRNA-seq	Bulk RNA-seq

* The sample size varies across different tissue types in GTEx v8, ranging from approximately 200 to 800 donors of European ancestry.

## Data Availability

All data generated or analyzed during this study are included in this published article and its Supplementary Information Files. Further inquiries can be directed to the corresponding author.

## Supplementary Materials

The following supporting information can be downloaded at https://www.mdpi.com/article/10.3390/genes17091097/s1: Figure S1: Overview of the csMR workflow; Figures S2–S6: Cell-type-stratified MR heatmaps for total cholesterol, VLDL-C, HDL-C, LDL-C, and triglycerides; Table S1: Colocalization results across lipid traits and brain cell types; Table S2: Final mediation analysis results for lipid–brain cell–plasma protein–kidney disease pathways; Table S3a–k: Conventional two-sample MR and cell-type-stratified MR results for total cholesterol, VLDL-C, HDL-C, LDL-C, and triglycerides; Table S4a–e: Sensitivity analyses for total cholesterol, VLDL-C, HDL-C, LDL-C, and triglycerides; Table S5: LDSC genetic correlation results between lipid traits and kidney outcomes; Table S6: Lipid–protein associations used for mediation pathway construction; Table S7: Dataset-level assessment of possible sample overlap among lipid GWAS, renal-outcome GWAS, and plasma-proteomic datasets.

## Abbreviations

The following abbreviations are used in this manuscript:

AgRP	Agouti-Related Peptide
ApoB	Apolipoprotein B
BBB	Blood–Brain Barrier
CGN	Chronic Glomerulonephritis
CKD	Chronic Kidney Disease
CNS	Central Nervous System
csMR:	cell-type-stratified Mendelian randomization
CTSF	Cathepsin F
DMV	Dorsal Motor Nucleus of the Vagus
eGFRE	Estimated Glomerular Filtration Rate
eQTL	Expression Quantitative Trait Loci
FDR	False Discovery Rate
GWAS	Genome-Wide Association Study
HDL-C	High-Density Lipoprotein Cholesterol
HMGCR	3-Hydroxy-3-Methylglutaryl-CoA Reductase
HPA	AxisHypothalamic-Pituitary-Adrenal Axis
ICAM4	Intercellular Adhesion Molecule 4
IV	Instrumental Variable
IVW	Inverse-Variance Weighted
LDL-C	Low-Density Lipoprotein Cholesterol
LDLR	Low-Density Lipoprotein Receptor
MR	Mendelian Randomization
NVU	Neurovascular Unit
ODCs	Oligodendrocytes
OPCs	Oligodendrocyte Precursor Cells
OR	Odds Ratio
OXT	Oxytocin
PPH4	Posterior Probability of Hypothesis 4
PVN	Paraventricular Nucleus of the Hypothalamus
RAS	Renin-Angiotensin System
ROS	Reactive Oxygen Species
SD	Standard Deviation
SNARE	Soluble N-ethylmaleimide-sensitive factor Attachment Protein Receptor
SNAP29	Synaptosome Associated Protein 29
SNS	Sympathetic Nervous System
TC	Total Cholesterol
TG	Triglycerides
TIMD4	T-cell Immunoglobulin and Mucin Domain Containing 4
TP53I3	Tumor Protein P53 Inducible Protein 3
UKB PPP	UK Biobank Pharma Proteomics Project
VLDL	Very-Low-Density Lipoprotein
VLDLR	Very-Low-Density Lipoprotein Receptor
